# Proteomic study uncovers molecular principles of single-cell-level phenotypic heterogeneity in lipid storage of *Nannochloropsis oceanica*

**DOI:** 10.1186/s13068-019-1361-7

**Published:** 2019-02-04

**Authors:** Chaoyun Chen, Andreas Harst, Wuxin You, Jian Xu, Kang Ning, Ansgar Poetsch

**Affiliations:** 1 Key Laboratory of Molecular Biophysics of the Ministry of Education, Hubei Key Laboratory of Bioinformatics and Molecular-imaging, Department of Bioinformatics and Systems Biology, College of Life Science and Technology, Huazhong University of Science and Technology, Wuhan, 430074 Hubei China; 2 Plant Biochemistry, Ruhr University Bochum, 44801 Bochum, Germany; 3 School of Biomedical and Healthcare Sciences, Plymouth University, Plymouth, PL4 8AA UK; 4 Single-Cell Center, CAS Key Laboratory of Biofuels and Shandong Key Laboratory of Energy Genetics, Qingdao Institute of BioEnergy and Bioprocess Technology, Chinese Academy of Sciences, Qingdao, 266101 Shandong China

**Keywords:** Single-cell, Phenotypic heterogeneity, Proteomics, Lipid storage, Cell sorting, *Nannochloropsis oceanica*

## Abstract

**Background:**

*Nannochloropsis oceanica* belongs to a large group of photoautotrophic eukaryotic organisms that play important roles in fixation and cycling of atmospheric CO_2_. Its capability of storing solar energy and carbon dioxide in the form of triacylglycerol (TAG) of up to 60% of total weight under nitrogen deprivation stress sparked interest in its use for biofuel production. Phenotypes varying in lipid accumulation among an *N. oceanica* population can be disclosed by single-cell analysis/sorting using fluorescence-activated cell sorting (FACS); yet the phenomenon of single cell heterogeneity in an algae population remains to be fully understood at the molecular level. In this study, combination of FACS and proteomics was used for identification, quantification and differentiation of these heterogeneities on the molecular level.

**Results:**

For *N. oceanica* cultivated under nitrogen deplete (−N) and replete (+N) conditions, two groups differing in lipid content were distinguished. These differentiations could be recognized on the population as well as the single-cell levels; proteomics uncovered alterations in carbon fixation and flux, photosynthetic machinery, lipid storage and turnover in the populations. Although heterogeneity patterns have been affected by nitrogen supply and cultivation conditions of the *N. oceanica* populations, differentiation itself seems to be very robust against these factors: cultivation under +N, −N, in shaker bottles, and in a photo-bioreactor all split into two subpopulations. Intriguingly, population heterogeneity resumed after subpopulations were separately recultivated for a second round, refuting the possible development of genetic heterogeneity in the course of sorting and cultivation.

**Conclusions:**

This work illustrates for the first time the feasibility of combining FACS and (prote)-omics for mechanistic understanding of phenotypic heterogeneity in lipid-producing microalgae. Such combinatorial method can facilitate molecular breeding and design of bioprocesses.

**Electronic supplementary material:**

The online version of this article (10.1186/s13068-019-1361-7) contains supplementary material, which is available to authorized users.

## Introduction

Population heterogeneity is a common phenomenon in microbial populations. For instance, genetic heterogeneity introduced by mutations is best understood in context of evolutionary adaptation. In recent years, it has been appreciated that certain environmental conditions as well as fluctuations in the microenvironment can elicit phenotypic heterogeneity in isogenic microbial cultures. Phenotypic heterogeneity occurs under conditions relevant to biotechnological processes, stress adaptation and biofilm formation: after continuous fed batch cultivation, an *E. coli* culture was found to consist of three subpopulations, one containing healthy cells, one containing cells with permeabilized membranes and dead cells [1, 2]. Cannibalistic subpopulations triggered by nutrient limitation were identified in *Bacillus subtilis* stationary phase cultures [3]. Furthermore, phenotypic heterogeneity plays an important role in the formation and migration of pathogenic *S. aureus* biofilms by emergence of two heterogeneous microcolony types with different metabolic profiles growing at different rates [4]. For valine-producing *C. glutamicum* cells, phenotypic heterogeneity in regard to their valine production was reported using a fluorescent reporter protein in microfluidic experiments; also population heterogeneity was identified concerning the activation of the CGP3 prophage [5, 6].

Analysis of population heterogeneity calls for methods allowing interrogation of features of interest on the single-cell level by microscopic or microspectroscopic methods. Of particular interest for biotechnology are methods that can be used to determine phenotypes where metabolite productivity can be monitored by fluorescence reporters [7]. Combined with high-throughput cell sorting methods, fluorescent features are used to differentiate heterogeneous populations for subsequent molecular analysis to unravel the mechanisms responsible for heterogeneity. Most prominent cell-sorting method is flow cytometry, FACS. The successful application of FACS for sorting of microbial populations has been reported in many publications, e.g., for *C. glutamicum* [8]; *E. coli* [9]; *P. putida* [10], and a microbial community [11].

*Nannochloropsis oceanica* is a photosynthetic unicellular microalga belonging to the eustigmatophyceae of the heterokont superphylum [12]. Its size ranges from 2 to 5 µm and its habitats include marine, fresh and brackish waters. Its ability to produce different fatty acid species was acknowledged in the late 1980s [13]. Its tremendous potential to accumulate lipid to a content of up to 60% of weight makes it an interesting organism for biotechnology [14]. To understand the processes leading to lipid accumulation, a number of OMICS studies have been performed: in 2014, the changes of the TAG synthesis pathway during nitrogen limitation were analyzed using transcriptomics and lipidomics [15]. The down-regulation of the Calvin cycle and the plastidic glycolysis pathway were reported by the transcriptomic analysis, while the tricarboxylic acid (TCA) cycle and pathways synthesizing pyruvate were upregulated in nitrate-deprived *N. oceanica* cells. Furthermore, an increase in TAGs was characterized by lipidomics during nitrogen deprivation where all TAG species were upregulated [16]. To compare nitrogen deprivation with nitrogen recovery, the proteome was analyzed in one study from 2013 [12], detecting 1500 protein spots using a two-dimensional polyacrylamide gel electrophoresis (2D-PAGE) gel from which 32 proteins showed differential expression and could be functionally annotated. Most prominent changes for nitrogen deprivation were decreased abundance of the putative Rubisco-regulator Calvin–Benson–Bassham cycle-related enzyme (cbbx), and one enoyl-acyl carrier protein reductase (enoyl-ACP reductase), whereas enzymes of nitrogen repletion assimilation, vacuolar proton pumps, and another enoyl-ACP reductase increased. Of note, due to the technical limitations of 2D-PAGE, detection of changes in membrane and basic proteins may have failed. In summary, despite lacking comprehensive proteomics, OMICS studies have been invaluable for the elucidation of physiological changes during nitrogen deprivation in *N. oceanica*.

The metabolic status of single cells in cultures of *N. oceanica* can differ in lipid content as uncovered by Raman spectroscopy, where single cells from the same condition showed high variation in regards to their hydrocarbon content [16]. Alternatively, analysis of lipid content on the single-cell level is feasible with fluorescent Nile Red staining, which was utilized in several studies with *N. oceanica* to sort strains with increased lipid content using FACS [17, 18]. The use of Nile Red to assess the lipid content in microalgal cells has become a standard method with a number of papers to support its capability to disclose single-cell heterogeneities [19], e.g., in *B. braunii*, *C. vulgaris* and *Chlamydomonas* sp.

Heterogeneity in the lipid content was elucidated for single cells in alginate hydrogel microcapsules [20]. Population heterogeneity in lipid content was also analyzed using FACS and BODIPY staining in *Cyclotella cryptica,* revealing the existence of subpopulations in regards to lipid content during nitrogen starvation, which, according to speculation of the authors, could be caused by epigenetic changes [21]. Even though molecular analysis of subpopulations could provide mechanistic explanation for population differentiation, it has not been attempted for any lipid-accumulating microalgae.

In this study, we observed population heterogeneity, manifested by differences in cellular lipid content, for *N. oceanica* cultivated under nitrogen replete (+N) and deplete (−N) conditions, for cultures grown in Erlenmeyer flasks and in a small-scale photo bioreactor. Quantitative proteomics was used to determine changes in metabolism that could mechanistically explain this outcome. Recultivation of sorted subpopulations and lipid content detection were pursued to verify a genetic cause of heterogeneity and to recognize the patterns based on which the phenotypic heterogeneity might follow.

## Materials and methods

### Precultivation of *N. oceanica* in f/2 media

*Nannochloropsis oceanica* IMET1 was inoculated on f/2 agar plates and grown for 6 days in darkness at 25 °C and then grown for 2 weeks at 50-µE white light until green colonies of *N. oceanica* were visible. Usually, one f/2 agar plate has 50–100 colonies.

Single clones from these colonies were inoculated in 100-ml modified liquid f/2 medium made with aquarium sea salt (Real Ocean, USA) supplemented with NaNO_3_ at 1 g/l as only nitrogen source. Cells were grown under continuous white light (roughly 80 ± 5 μmol photons/m^2^ s = µEinstein) at room temperature (RT) and aerated by bubbling with compressed air in the 250-ml flask. Cultures were grown until they reached the mid-exponential phase (optical density at 750 nm (OD750) of 5.0 ± 0.5). OD750 was measured using appropriate dilution in isometric germ-free sodium chloride solution. To check for bacterial contamination, 90 µl was taken from each culture and mixed with 10 µl 0.01 µg/µl DAPI (Solarbio C0060) [22]. Mixtures were incubated in dark for 20 min, then analyzed with a fluorescence microscope and laser Ex 300–385 nm, Em 420 nm and dichroism at 400 nm. Bacteria stained by DAPI appear blue in field of vision, whereas algae due to chloroplast autofluorescence turn red.

### Main cultivation in Erlenmeyer flasks

Appropriate cultures were taken from the preculture medium and diluted to OD750 of 1 in 100 ml; for +N, modified liquid f/2 medium with NaNO_3_ at 1 g/l, and for –N. only modified liquid f/2 medium was used. Each culture was transferred to a 250-ml flask and grown for 2 weeks at 50 µE, 25 °C [23] until OD750 of about 5 was reached and shaken once per day, before sorting of the two subpopulations in the sample. Each week, the medium was replaced with fresh one.

### Recultivation in Erlenmeyer flasks

For the recultivation experiment to investigate stability of heterogeneity in the *N. oceanica* IMET1 cultures, FACS-sorted +N subpopulations were grown in modified liquid f/2 medium with 1 g/L NaNO_3_. To inoculate medium, 4 million cells of each +N P3 and +N P4 population (defined in “FACS analysis and cell sorting of *N. oceanica* cells”) were sorted onto a sterile 0.22-µm filter cartridge. Filter material containing the cells was extracted from the filter casing in the clean bench; the extracted filters [23] were then transferred to 50 ml +N f/2 medium in a 250-ml flask and grown for 40 days in a light cupboard with 50-µE light at 25 °C. Flasks were daily checked for growth and shaken. After cultures had reached an OD750 value of 1, filter membrane fragments were separated by gravitational precipitation overnight. The supernatant was centrifuged at 6800*g* for 10 min at room temperature (RT) and the cell pellet was resuspended in 200 ml +N f/2 and cultivated in 500-ml flasks as described in main cultivation in Erlenmeyer flasks until OD750 of 5 was reached in 2 weeks.

### Main cultivation in small-scale photo bioreactor

Cells from preculture were injected to OD750 value of 0.1, and cultures grown in two 1-L glass columns (inner diameter: 5 cm) containing 600 ml +N f/2 medium until mid-exponential phase with OD750 = 5 ± 0.5, room temperature was 25 °C with 80 ± 5 μmol/m^2^ s continuous white light irradiation. Cultures were bubbled with compressed air. After 2-week culture, *Nannochloropsis* cultivations were individually transferred into centrifuge tubes and centrifuged at 6800*g* for 10 min at RT (HITACHI, centrifuge R9A rotor). Algae pellets were washed twice with 100-ml nitrogen-deficiency f/2 medium (f/2 medium without sodium nitrate). After centrifugation (Beckman Coulter Allegra X-12R, SX4750 Rotor, 2000*g*, 10 min) pellets from three cultures were resuspended with 600-ml modified f/2 medium containing 1 g/l NaNO_3_ and pellets from the other three replicates were resuspended with 600-ml nitrogen-deficient f/2 medium. After 10 days of cultivation, cells were harvested from the cultures under a clean bench. Each cultivation was divided into three sections corresponding to the top, middle and bottom parts of the reactor.

### Light microscope-based cell counting

Microscopic cell counting of *N. oceanica* cells was performed using a Thoma cell counting chamber. Cells were counted in the c-fields of one b-field of the Thoma cell counting chamber with a 40× objective. All c-fields in a b-field were counted. All c-fields were averaged over the four b-fields. The average number of cells for all c-fields was multiplied by 4 million to obtain the number of cells per milliliter.

### Lipid staining of *N. oceanica* cells

Nile Red staining of neutral lipids was used to sort individual *N. oceanica* cells and to observe heterogeneity with FACS according to their lipid content. For staining with Nile Red, *N. oceanica* cells were aliquoted in the required number as established by cell counting. The aliquoted cells were washed three times using 35 g/l sea salt water (2380*g*, 5 min, Eppendorf 5804, at RT) and then diluted to a cell concentration of 20 million cells per milliliter. First, the Nile Red stain was optimized with fluorescence microscopy to achieve highest Nile Red intensity for lipid-containing *N. oceanica* cells using fluorescence spectroscopy (Additional file 1: Figure S1). Accordingly, cells were diluted in a mixture of 0.9% sodium chloride with 15% DMSO. Nile Red was added to this solution in a volume ratio of 1:100 from a 219 µmol/l Nile Red stock in MeOH solution according to [17]. The samples were quickly vortexed and then stored in the dark for at least 10 min.

### FACS analysis and cell sorting of *N. oceanica* cells

To prove occurrence of population heterogeneity in *N.* oceanica, single-cell analysis with FACS was chosen. Sorting of *N. oceanica* cells was performed using a MoFlo™ XDP High-speed cell sorter. Before cell sorting, beads (Flow-Check™ Fluorospheres 6605359, Beckman Coulter, Inc. USA) were used for fine alignment of the instrument. FL2-Log-Height fluorescence channel was applied for Nile Red fluorescent intensity detection. FSC-Height, FSC-Width and SSC-Height, presenting the morphological features of the input particles here, were used to measure the *N.* oceanica cells. For acquisition of results from cell-sorting experiment, the sorting protocol of the MoFlo™ XDP High-speed cell sorter was set to consist of three histograms: in the first histogram holding a gate R1, signals lower than 128 in FSC-Height and lower than 224 in SSC-Height were filtered out; in the second histogram, a spindly rectangle gate R2 was applied in the FSC-Height to FSC-Width histogram to remove cells which were not single; then in the last histogram, the target single cells were sorted, so that single cells for one subpopulation would be sorted to gate R3 (referred to as subpopulation P3), and another subpopulation would be sorted to gate R4 (referred to as subpopulation P4). For –N *N. oceanica* cells, the two sorted subpopulations are referred to as −N P3 and −N P4; For +N *N. oceanica* cells, the two sorted subpopulations are referred to as +N P3 and +N P4.

After Nile Red addition, cells in the staining solution were diluted to a cell concentration of 20 million cells per ml using 0.9% sodium chloride which was filtered with a 0.22-µm filter. Subpopulations were identified by the SSC channel filtering at 488 nm with a 10-nm bandpass and the Nile Red fluorescence channel filtering at 580 nm with a bandpass filter, filtering out the signal with wavelength below 500 nm and above 580 nm. Droplet formation was optimized and drop delay was determined using the Drop Delay Wizard. The sorting process was controlled by the Summit software 5.5.0.16880 (Beckman Coulter, Inc.). 10,000,000 cells of each subpopulation were sorted into 30 ml of Puraflow 8× Sheath Fluid, and cells were stored at − 80 °C and then lyophilized using an ultradry lyophilizator to obtain dried samples that were shipped from China to Germany for proteome analysis.

### Digestion of proteins on filter well plates

The lyophilized cells were concentrated on 96 filter well plates by resuspension in 15-ml MilliQ water per falcon tube and the solution was homogenized by short vortexing. 300-µl portions of this solution were applied to each well of the 96-well filter plate (Millipore MSGVS2210) and the liquid was aspirated using a 96-well plate table top aspirator (KNF Neuberger Laboport). To increase the speed of the aspiration process, each subpopulation was aspirated in three separate wells; after aspiration, membranes with cells were washed with MilliQ grade water for three times.

The digestion protocol used for *N. oceanica* was based on [24]. Filter membranes containing cells from the same sample were first collected in one tube and then divided into smaller pieces by a scalpel and dissolved in 32-μl dissolution buffer (25-mM ammonium bicarbonate, pH 7.8 containing 2-µl acetonitrile). Subsequently, cells were proteolytically digested with 8-μl porcine trypsin (Promega, Mannheim, Germany) resulting in a working concentration of 0.25 μg/μl at 37 °C with continuous shaking at 400 rpm for 2 h. Afterwards, cell debris and filter membranes were removed by centrifugation at 13,000*g* for 10 min at RT. Supernatants were collected in a glass vial and vacuum concentrated until the glass vial was dry. Dried peptides were resuspended in buffer A (2% acetonitrile 0.1% formic acid) by sonification for 5 min in a water bath. After centrifugation (2 min at 380*g* RT), samples were measured with mass spectrometry (MS).

### Protein identification via 1D-nLC-ESI–MS

Protein identification was conducted in the same way as described in [8]. Measurements were performed using a nanoAcquity UltraPerformance LC system connected to an auto-sampler equipped with a HSS T3 analytical column (1.8-µm particle, 75 µm × 150 mm) kept at 45 °C, and a Symmetry C18 trap column (5-µm particle, 180 µm × 20 mm), all Waters, USA. This setup was connected to an LTQ Orbitrap Elite. A 180-min gradient was used: (0–5 min: 99% buffer A and 1% buffer B, 5–10 min 99–94% A, 10–161 min: 94–60% A, 161–161.5 min: 60–14% A, 161.5–166.5 min: 14–4% A, 166.5–167.1 min: 99% A, 167.1–180 min: 99% A). To optimize the method for small cell numbers, the MS/MS max ion inject time was increased to 400 ms.

### MS data processing

The identified proteins from the *N. oceanica* samples were identified by the Andromeda algorithm embedded in the MaxQuant software version 1.5.5.1 [25]. All datasets were searched against an *N. oceanica* IMET1 database containing 9915 sequences [15]. Only tryptic peptides with minimum of seven amino acids length were considered for the identification of proteins. No fixed modifications were used in the searches, while oxidation of methionine and N-terminal acetylation were set as variable modifications. The mass tolerance for CID fragment ion matches was set to 0.5 Da. Proteins were quantified by label-free quantification (LFQ) integrated into the MaxQuant software, and used for quantification only, when they had two or more unique peptides [26]. Presented area values for *N. oceanica* are based on these quantification results. Significantly regulated proteins were established by a two-sample *t* test based on median normalized log2 abundance values of three replicates, and FDR (*q* value of ≤ 0.05) calculation by randomizing samples. The mass spectrometry proteomics data have been deposited to the ProteomeXchange Consortium via the PRIDE [27] partner repository with the dataset identifier PXD008721.

### Recultivation and reanalysis of subpopulations

For the purpose of recultivation and reanalysis, the subpopulations +N P3 and +N P4 were obtained by sorting exactly as described in previous sections. After 40 days, the recultivation reached a cell density sufficient for FACS sorting. Triplicate samples for each recultivated subpopulation (+N P3, +N P4) were used. Each sample for resorting was stained with the same staining method referred to in the previous sections. The protocol as referred to in section “FACS analysis and cell sorting of *N. oceanica*” was applied to detect the appearances of subpopulations in the sorted cells upon recultivation. After Nile Red staining, cells in the staining solution were diluted into the concentration of 20 million cells per milliliter using sodium chloride which was filtered with a 0.22-µm filter for sorting with a MoFlo™ XDP High-speed cell sorter in the purify sorting mode. 10,000 cells of each subpopulation were sorted into 30 ml of Puraflow 8× Sheath Fluid, for collecting the subpopulations’ features for further analysis.

### Statistical assessment of differences between cultivation conditions

To analyze the sample differences among samples based on all MS data acquired in this study, the relative abundances of co-occurring proteins were analyzed by principal component analysis (PCA) implemented in R (https://www.r-project.org/) with ade4 package [28].

## Results

### FACS sorting of +N and –N *N. oceanica* subpopulations

**Fig. 1:**
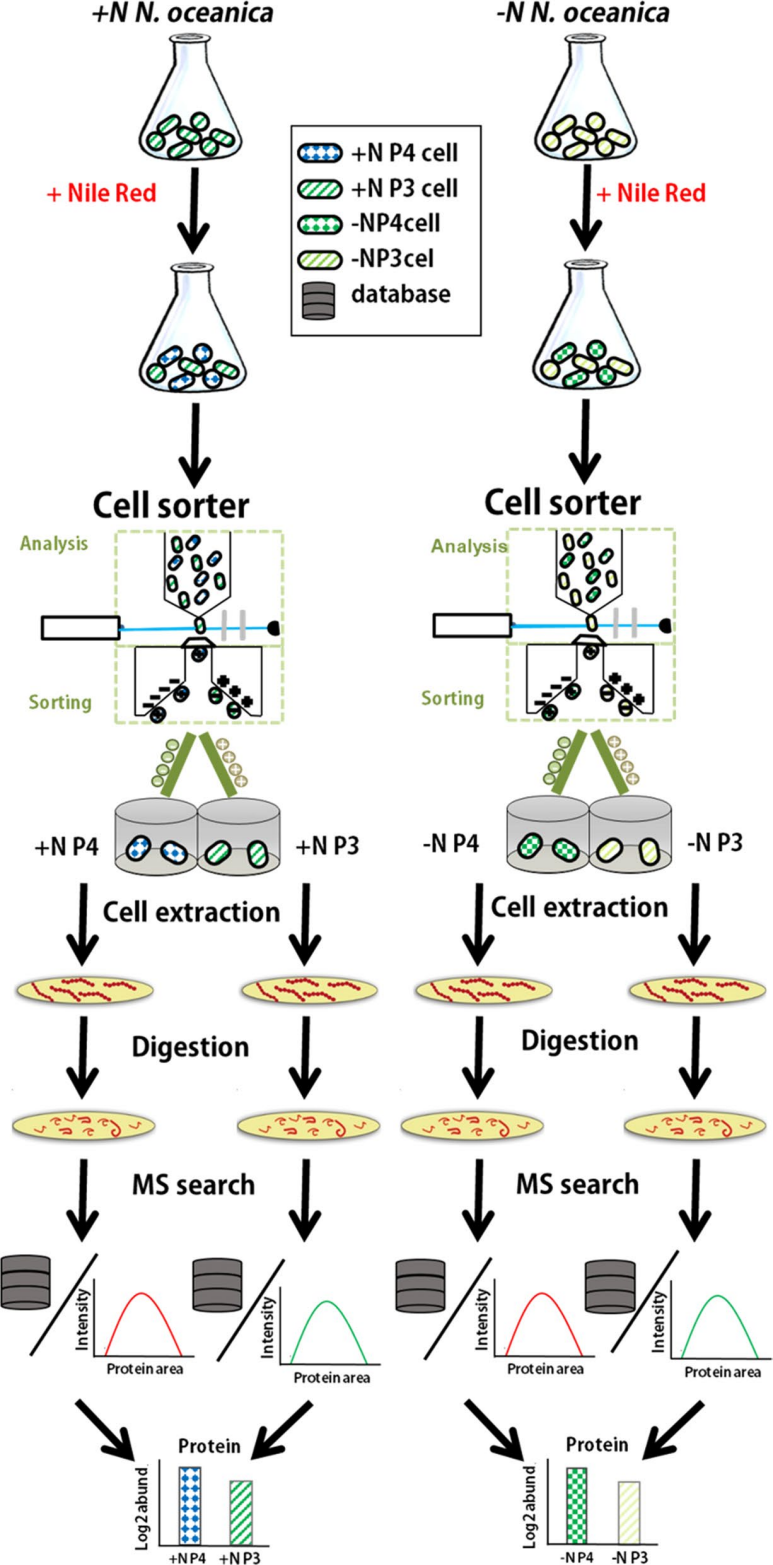
Workflow of FACS sorting and subsequent proteomic analysis of +N and –N *N. oceanica* subpopulations. After *N. oceanica* cells are stained with Nile Red for lipids, population heterogeneity can be detected by FACS-based cell sorting (+N P4 cells (blue diamonds); +N P3 cells (dark green diagonal lines); −N P4 cells (dark green chessboard); −N P3 cells (light green diagonal lines). Subsequent to sorting, cells are concentrated in 96 filter well plates, on which also cell lysis takes place. The extracted proteins are then digested by trypsin on the membrane. Data generated by mass spectrometry (MS) of these peptides are searched against a sequence database of *N. oceanica* proteins. Identified proteins are then quantified by LFQ algorithm of MaxQuant, to determine differences in proteome composition

**Fig. 2:**
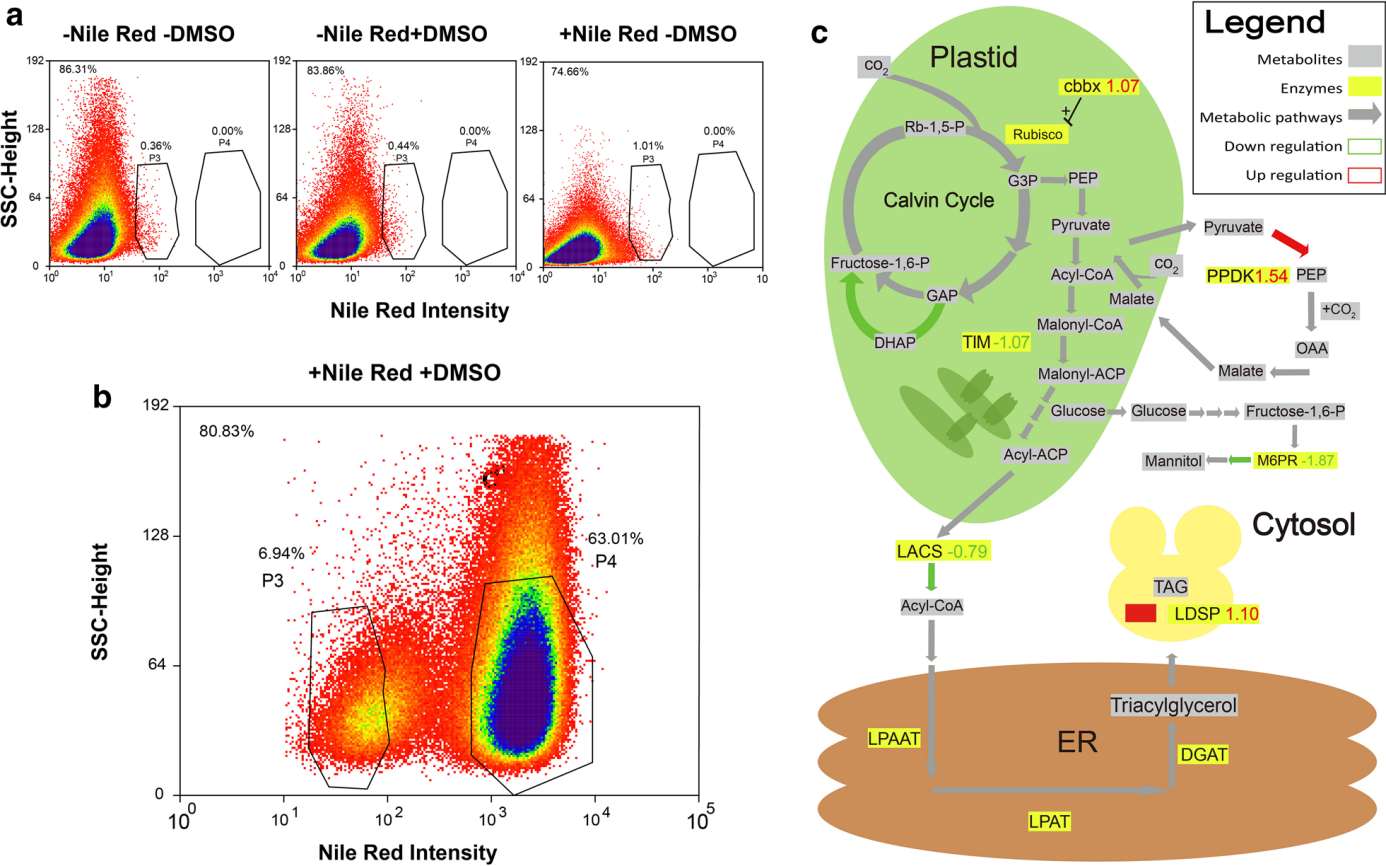
FACS sorting of +N *N. oceanica* cultures and proteomic abundance differences on triacylglycerol pathway. **a** Autofluorescence control for unstained +N *N. oceanica* cells, control for cells which were incubated in 15% DMSO and control cells incubated with 0.7 µg/ml Nile Red without DMSO. **b** Cell density plot +N *N. oceanica* cells stained with Nile Red, the SSC (Sidescatter)-height channel informs about cell size and morphological features, while the Nile Red intensity axis value reports the intensity of Nile Red fluorescence detected through FL2-Log-Height fluorescence channel. Percentage in upper left corner indicates percentage of depicted events from total. Percentages above subpopulations denote gated cell fraction from total. **c** Diagram of regulation of enzymes belonging to the carbon fixation towards triacylglycerol pathway between +N P3 and +N P4 subpopulations. Metabolites are highlighted by gray boxes and enzymes are highlighted by yellow boxes, color of the arrows indicates up- or down-regulation, while the numbers adjacent to the enzyme names are the regulation factors (log2). Rb-1,5-P: Ribulose-1,5-bisphosphate; RUBISCO: ribulose-1,5-bisphosphate carboxylase; cbbx: cbbx homolog to protein activating expression of Rubisco; G3P: 3-phospho glycerate; F-1,6-P: fructose-1,6-bisphosphate; GAP: glycerine aldehyde-3-phosphate; DHAP: dihydroxyacetone-phosphate; PEP: phosphoenolpyruvate; Malonyl-CoA: Malonyl-coezyme A; Malonyl-ACP: Malonyl-acyl carrier protein; Acyl-CoA: acyl-coenzyme A; LACS: Long chain acyl CoA synthetase; LPAAT: lysophosphatidic acid acyltransferase; LPAT: lyso-phosphatidylcholine acyltransferase; DGAT: diacylglycerol acetyltransferase; ER: endoplasmic reticulum; OAA: oxaloacetate; TAG: triacylglycerol; LDSP: lipid droplet surface protein; M6PR: NADPH dependent Mannose-6-phosphate reductase; PEP: phosphoenolpyruvate; OAA: oxaloacetate; PPDK: pyruvate, phosphate dikinase; TIM: triosephosphate isomerase. CO_2_ fixation by Rubisco and fatty acid biosynthesis occurs in plastid, carbon fixation involving PPDK in cytosol, TAG synthesis in ER and storage of TAG in lipid bodies (filled yellow)

FACS control experiments were performed to check for autofluorescence, population separation and artifacts. FACS analyses were conducted as depicted in Fig. 1, and the FACS analysis results of +N *N. oceanica* cells in Fig. 2a revealed no subpopulations in unstained cells, as well as for cells prepared in 15% DMSO without Nile Red. Without staining, only small variety of intensities in the Nile Red channel filtering at 575 nm was present, which was regarded as background fluorescence (Fig. 2a). This trend was continued in the Nile Red-stained *N. oceanica* cells prepared without DMSO, where also only background fluorescence was recorded (Fig. 2a). Samples stained by Nile Red with 15% DMSO reached higher fluorescence intensity under identical measurement conditions and thus, this staining protocol was considered as the most efficient staining procedure for lipid-containing cells.

Intriguingly, the Nile Red signals of *N. oceanica* were distinguished into two subpopulations as shown in (Fig. 2b); thus, strong population heterogeneity in *N. oceanica* under +N conditions was detected. The sorted subpopulations +N P3 and +N P4 exhibited different fluorescence intensity in the Nile Red height channel in (Fig. 2b). As Nile Red intensity is an indicator of neutral lipid content [19], it can be concluded that +N P4 cells contain more lipid than +N P3.

**Fig. 3:**
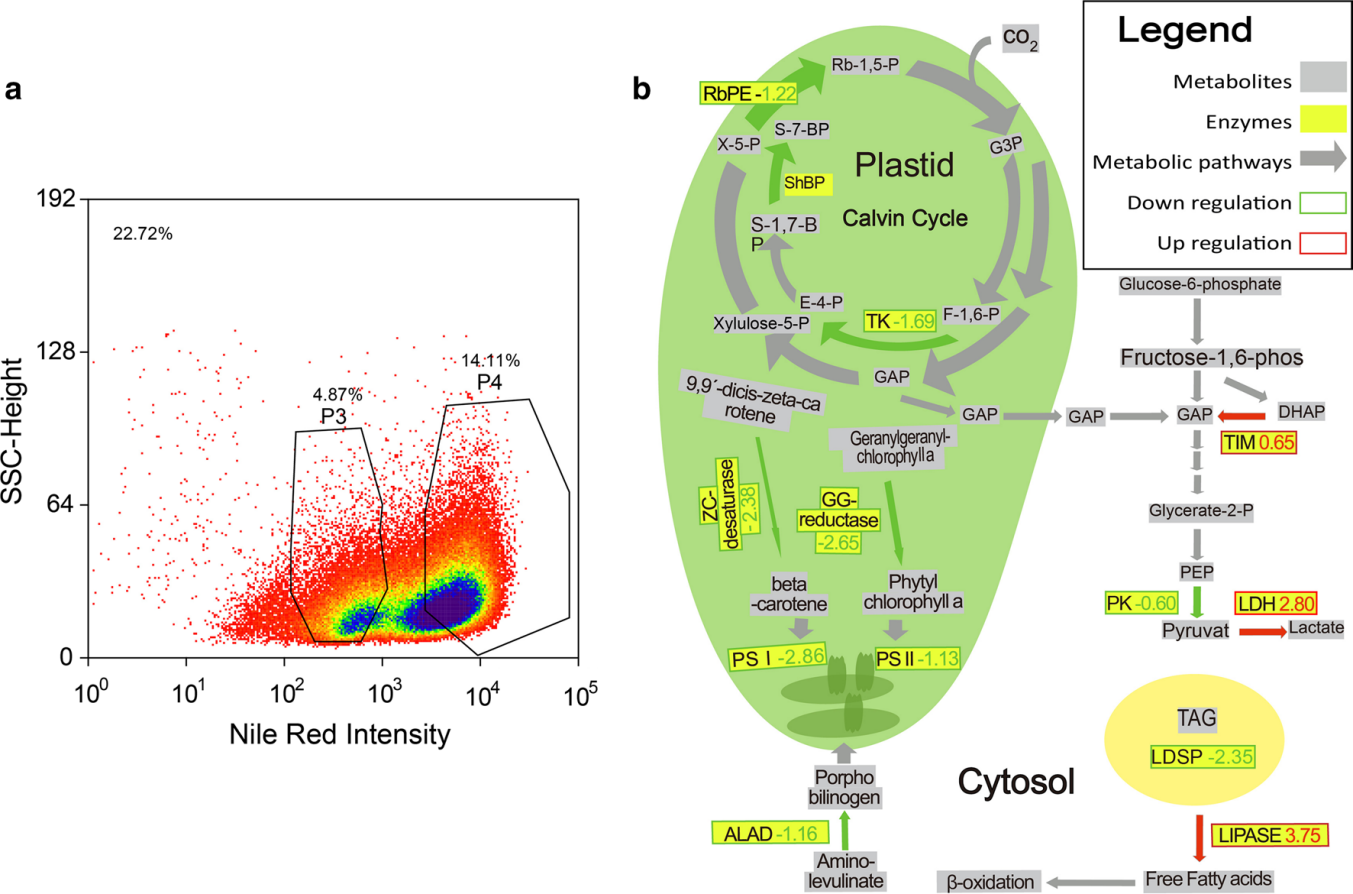
FACS sorting of –N *N. oceanica* cultures and proteomic abundance differences on carbon fixation pathway. **a** Cell density plot –N *N. oceanica* cells stained with Nile Red, the SSC (Sidescatter)-height channel informs about cell size and morphology, while the Nile Red Intensity reports the intensity of the FL2-Log-Height fluorescence channel. In this case, two subpopulations exist with one showing increased Nile Red fluorescence intensity in the FL2-Log-Height fluorescence channel. Percentage in upper left corner indicates percentage of depicted events from total. Percentages above subpopulations denote gated cell fraction from total. **b** Diagram of regulation of enzymes belonging to the carbon fixation pathway as well as to the glycolysis pathway between −N P3 and the −N P4 subpopulation. Significant regulation between subpopulations was calculated by a two-sample *t* test with a FDR cutoff by 0.05. Metabolites are highlighted by gray boxes and enzymes are highlighted by yellow boxes, color of the arrows shows upregulation or downregulation, while the numbers adjacent to the enzyme names are the regulation factors. Rb-1,5-P: ribulose-1,5-bisphosphate; G3P: 3-phospho glycerate; F-1,6-P: fructose-1,6-bisphosphate; TK: transketolase; GAP: glycerine aldehyde-3-phosphate; E-4-P: erythrose-4-phosphate; S-1,7-BP: sedoheptulose-1,7-bisphosphate; ShBP: sedoheptulose bisphosphatase; S-7-P: sedoheptulose-7-phosphate; X-5-P: xylulose-5-phosphate; RbPE: ribulose phosphate epimerase; ZC-desaturase: zeta-carotene desaturase; GG-reductase: geranylgeranyl-reductase; PSI: photosystem I; PSII: photosystem I; ALAD: aminolevulinic acid dehydratase; G-6-P: glucose-6-phosphate; DHAP: dihydroxyacetone-phosphate; TIM: triosephosphate isomerase; PEP: phosphoenolpyruvate; LDH: lactate dehydrogenase; PK: pyruvate kinase; TAG: triacylglycerol; LDSP: lipid droplet surface protein; ER: endoplasmic reticulum. CO_2_ fixation in Calvin cycle involving RbPE, ShBP and TK occurs in plastid, pigment biosynthesis involving ZC-desaturase and GC-reductase occurs in plastid. Energy production from carbon via glucose-6-phosphate to pyruvate and lactate involving TIM, PK and LDH occurs in cytosol. Storage and release of fatty acids in lipid droplet (yellow) involves LDSP and lipase

After establishing +N population heterogeneity, a similar investigation of –N *N. oceanica* followed. With the same staining process as for +N *N. oceanica*, the –N *N. oceanica* cells also displayed population heterogeneity (Fig. 3a). Thus, −N and +N both displayed a split into two populations differing in Nile Red intensity. To obtain molecular details that explain the differences in lipid accumulation, proteome analysis was performed with the same method for +N *N. oceanica* and –N *N. oceanica.* Albeit SSC vs Nile Red intensity was chosen for gating of subpopulations, their separation was obtained with FSC vs Nile Red as well (Additional file 1: Figure S2c, d). However, FSC vs SSC did not show such separation, indicating that no distinct difference exists between cell size and morphology for the subpopulations (Additional file 1: Figure S2a, b).

### Fluorescence light microscopy of *N. oceanica* +N and −N cells

Laser scanning confocal microscopy (LCSM) as described in Additional file 1: M&M was used to confirm the presence of populations with differing lipid content for *N. oceanica* cells cultivated in +N or −N medium. Furthermore, to assess selective staining of neutral lipids by Nile Red, it was compared to BODIPY 505/515 stain. As depicted in Additional file 1: Figure S3a, staining results appear very similar. Overall, nitrogen deprivation leads to increased size of liposomes and decreased size of chloroplast, exemplified in Additional file 1: Figure S3b. Concerning population heterogeneity, microscopic analysis is in line with FACS; N− and N+ cultivation contains cells with relatively high and relatively low lipid content (Additional file 1: Figure S3c). Regarding chloroplast structure and chlorophyll content, another level of heterogeneity is evident and displayed for selected single cells, i.e., B vs A + C and D vs E + F in Additional file 1: Figure S3c.

#### Proteomics of FACS-sorted +N *N. oceanica* subpopulations

Three independent cultivations of +N *N. oceanica* were sorted with FACS to obtain subpopulation +N P3 and +N P4, respectively. Using the workflow depicted in Fig. 1, 1424 proteins were identified and quantified in the subpopulation proteome datasets +N P3 and +N P4 (P4 containing more lipids and P3 containing less lipids); this translates into 14% coverage of the theoretical proteome. Of all these proteins, only 42 were considered as being significantly different between subpopulations with an FDR ≤ 0.05 as depicted in a volcano plot in (Additional file 1: Figure S4a).

Among the 42 significantly regulated proteins presented in Additional file 1: Table S1 covering various cellular processes, here focus is on lipid production and steering carbon flux as shown in (Fig. 2c). The gluconeogenetic enzyme pyruvate orthophosphate dikinase (PPDK), which in higher plants is important for the C4 cycle, showed strong upregulation (1.54) as well as the cbbx protein activating the ribulose-1,5-bisphosphate carboxylase (RUBISCO) (1.07). On the other hand, the enzymes triosephosphate isomerase (TIM) (− 1.07) and long-chain acyl CoA synthetase (LACS) (− 0.79) were downregulated; the lipid droplet surface protein is involved in the formation of lipid droplets and its abundance (1.1) was increased in subpopulation +N P4. Taken together, the data point to an increased production of lipids in +N P4 in accordance with the higher lipid content detected with Nile Red. Enzymes which are not directly connected to the carbon metabolism were also significantly regulated: NADPH-dependent Mannose-6-phosphate reductase (M6PR) (− 1.87), which takes part in the synthesis of mannitol, was downregulated in +N P4. The chlorophyll protein fucoxanthin showed decreased abundance (−0.21), as well as the enzyme isopropyl malate dehydrogenase involved in isoleucine biosynthesis (−0.40).

#### Proteomics of FACS-sorted −N *N. oceanica* subpopulations

Three independent cultivations of –N *N. oceanica* were sorted with FACS to obtain subpopulations −N P3 and −N P4 and further processed in an equal manner as +N samples. For the −N subpopulations, in total 1264 proteins could be identified of which 156 proteins were significantly regulated between −N P3 and −N P4 (volcano plot in Additional file 1: Figure S4a).

In the −N P4 subpopulation, Calvin cycle enzymes were downregulated while glycolysis was upregulated (Fig. 3b). Enzymes of carbon fixation being part of the Calvin cycle, such as sedoheptulose bisphosphatase (− 1.52), ribulose phosphate epimerase (− 1.22), and transketolase (− 1.69) were downregulated. On the other hand, enzymes of energy producing pathways, d-lactate dehydrogenase (2.80) and triosephosphate isomerase (0.65), were upregulated, whereas pyruvate kinase (− 0.60) was downregulated. While the lipid droplet surface protein (− 2.35) was downregulated, the putative lipase (3.75) and lysophospholipase II (1.72) were strongly upregulated. Furthermore, a number of proteins of the photosynthetic apparatus were downregulated, for example, several proteins of the photosystem I (− 2.86) and photosystem II (− 1.13), while the photosystem II oxygen evolving complex (1.98) was upregulated. Enzymes which synthesize precursors for light harvesting carotenoids were also downregulated, for instance, geranylgeranyl reductase (− 2.65), zeta-carotene desaturase (− 2.38), and delta-amino levulinic acid dehydratase (− 1.16); additionally, light-harvesting proteins (− 1.84) saw downregulation. In general, here downregulation of carbon fixation by the Calvin cycle and degradation of the photosynthetic apparatus can be reported, while parts of pathways producing energy from carbon were upregulated. Aside from proteins related to carbon metabolism and photosynthesis, various other cellular functions were affected, e.g., cellular stress and protein folding, polyketide synthesis, histones (see Additional file 1: Table S2).

#### Comparison of −N and +N subpopulations

To verify, if subpopulations showed resemblance on the molecular level between +N and −N cultivation, comparison of the −N subpopulation proteomes with the +N subpopulation proteomes based on PCA was performed (Additional file 1: Figure S4b). Results demonstrated a clear-cut difference between −N and +N subpopulations with the first principal component at a value of 83.13%. Furthermore, the +N subpopulation replicates did not have strong variation between the proteomes, while especially the −N P4 proteomes displayed strong variation. Clear difference between −N and +N subpopulations observed by PCA is reflected by comparing all proteomes using *t* test, where a major difference between +N and −N subpopulations can be found: around 493 significantly regulated proteins could be identified between +N P4 and the combined proteomes of −N P3 and −N P4, and about 267 proteins could be identified for +N P3 and combined −N P3 and −N P4 subpopulations. Significant regulation between +N and −N subpopulations was found for in total 430 proteins with a *p* value below 0.05, including important enzymes as phosphoglycerate mutase, glyceraldehyde 3 phosphate dehydrogenases, triosephosphate isomerase, pyruvate phosphate dikinase, phospho-glycerate kinase, and ribulose phosphate-3-epimerase (Additional file 1: Table S3).

To compare the lipid content between the subpopulations, as well as the lipid content between different culture conditions in this study, the averaged Nile Red intensity of each subpopulation was calculated, with the value of 576.34, 6534.17, 67.27, 1770.47 for the −N P3, −N P4, +N P3, +N P4 subpopulation, respectively. The absolute cell numbers used for calculation are presented in the Additional file 1: Figure S5c) for +N P3 and +N P4, and Additional file 1: Figure S5d) for −N P3 and −N P4. Thus, the relative lipid content ratios were determined as follows: +N P4 to +N P3 is about 238.96 (defined as relative lipid content of +N P4 divided by relative lipid content of +N P3), −N P4 to −N P3 is about 32.85 (defined as relative lipid content of −N P4 divided by relative lipid content of −N P3), and −N to +N is about 3.13 (defined as relative lipid content of −N cells divided by relative lipid content of +N cells).

### Recultivation and reanalysis for +N subpopulations

To establish the nature of the observed heterogeneity (genotypic vs phenotypic) recultivation experiments were performed for +N *N. oceanica*. Recultivated for 40 days, the cultivation suspension of the +N P3 and +N P4 displayed a green color similar to the first round of +N *N. oceanica* cultivation, and reached a high cell concentration to averaged OD750 value of 5.96 for +N P3 recultivation and averaged OD750 value of 6.45 for +N P4 recultivation. Upon staining with Nile Red in DMSO for 10 min in darkness, the samples were analyzed with MoFlo™ XDP High-speed cell sorter. The FACS results demonstrated the same population heterogeneity for cells from +N P3 and +N P4 recultivations (Additional file 1: Figure S6). In the three recultivated replicates of +N P3 and +N P4, each showed the occurrence of two subpopulations though their respective densities differed from first round. This indicates that population heterogeneity reappears after sufficient cultivation time for both +N P3 and +N P4.

### Analysis of −N cells from small-scale bioreactor

To address the occurrence of the observed heterogeneity in a biotechnological cultivation process, a further cultivation was carried out in a small-scale photo-bioreactor under nitrogen deprivation. The cultures showed a strong color change from green in the +N f/2 cultivation phase to deep yellow after −N f/2 treatment on the 10th day as a response to nitrogen depletion. The 600-ml −N cultivations were divided into three different origins from the bioreactor (“bottom”, “middle”, “top”). For all parts of the bioreactor, subsequent FACS analysis with Nile Red was performed. The fluorescence intensity of unstained cells (not shown) showed a similar fluorescence pattern in all three parts of the bioreactor, where two subpopulations could be identified in the Nile Red-stained cultures (Additional file 1: Figure S7), whereas separation is less distinct than for Erlenmeyer flask cultivation. Thus, it can be concluded that population heterogeneity can occur in a photo bioreactor, too.

## Discussion

### Occurrence of population heterogeneity in lipid storage for *N. oceanica*

In this study, for the first time, proteomes of distinct *N. oceanica* subpopulations were analyzed upon single-cell sorting using FACS based on neutral lipid sensitive Nile Red staining. Analysis of cultivations differing in cellular lipid content by fluorescence microscopy and spectroscopy in Additional file 1: Figure S1 confirmed that the used Nile Red-staining conditions adapted from [17] inform about lipid content on the single-cell level. Indeed, the utility of Nile Red for sorting of single cells based on lipid content has been demonstrated previously for several algae, including *Nannochloropsis* sp., *Chlamydomonas reinhardtii* [29], and *Dunaliella salina* [30]. Here, we could discriminate subpopulations with differing lipid content for cells cultivated under nitrogen +N, as well as for −N conditions. Cells grown for more than 2 weeks, being already in stationary phase, were used for population heterogeneity detection. The phenomenon of heterogeneous intracellular lipid content in microalgae for various cultivation conditions and species is well documented. For +N and −N conditions, heterogeneity in TAG content was observed in *N. oceanica* with single cell Raman microscopy, though only up to 4-day-old cultivations were investigated [16]. By means of imaging cytometry, distinct *Cyclotella cryptica* subpopulations were revealed after 4 days of −N conditions, whereby the majority of cells could be classified as small-lipid containing, or TAG hyperaccumulating with high or low chlorophyll content, respectively [21]. In view of this study and our fluorescence microscopy data, also our *Nannochloropsis* cultivations actually consist of more than the two subpopulations selected solely for lipid accumulation with FACS. Occurrence of heterogeneity in lipid content after prolonged cultivation has been reported [18] too. *Nannochloropsis* sp. grown for 15 days under +N conditions showed small subpopulations with increased Nile Red fluorescence intensity. It is likely that high percentage of old age cells stored energy in the form of TAG; yet there was no analysis of the physiological status [17, 18].

FACS-based multiple selection rounds of subpopulations with high lipid content have successfully been exploited for the breeding of superior lipid producers. Though rounds of recultivation and FACS sorting are without a doubt an effective novel breeding approach, the causative chain of molecular events resulting in favorable traits remains enigmatic. For the successful cases addressing lipid production, authors implicitly assumed manifestation of genetic changes; yet it may well be that (some) cells in the first selection rounds only displayed a lipid-rich phenotype. Indeed, as discussed in more detail later, our results for one round of *N. oceanica* recultivation of sorted subpopulations refute genetic differences in the population. Consequently, one should be cautious to immediately attribute observed heterogeneity to genetic differences, as population heterogeneity could result from varying cultivation factors [31, 32]. Intriguingly, heterogeneity might also be a consequence of biological adaptation according to the fractal theory of new conditions, which is discussed later.

There is consensus that modern OMICS technologies are bespoke tools for addressing the exciting question of emergence and biotechnological utilization of population heterogeneity [33]. Being part of this arsenal, the mature quantitative proteomics developed for *N. oceanica* disclosed distinct metabolic differences between subpopulations with their functional consequences discussed in the following paragraphs.

### Differentiation in lipid metabolism for +N *N. oceanica* subpopulations

For the +N condition of 1424 identified and quantified proteins, only 42 were significantly regulated between subpopulations +N P3 and +N P4. Subpopulation +N P4 showed increased Nile Red fluorescence. The metabolism of this subpopulation, therefore, has adapted to the production of fatty acids as many significantly upregulated proteins and enzymes show. The most prominent was the upregulated lipid droplet protein (log2 1.10); in the diatom *Phaeodactylum tricornutum,* a homologue of this protein was identified as the most abundant protein in lipid droplets, substantiating its significance for the formation and stability of lipid droplets [34]. Additionally, the enzyme PPDK, which is involved in the C4 metabolism in higher plants, showed a strong upregulation (1.54) in +N P4. This could point to a carbon concentration mechanism formulated for *Thalassiosira pseudonana* [35]. To this pattern fits the upregulation of cbbx by 1.07, a protein which activates the red-type rubisco protein, the major incorporator of CO_2_ into biomass [36]. On the other hand, pathways competing for carbon flow were downregulated, e.g., Mannose-6-phosphate reductase catalyzing the last step in the pathway to production of mannitol in celery [37]. Additionally, enzymes such as LACS, which play an important role in transferring fatty acids from the plastid to the cytosol, were downregulated [38]; also, the TIM protein involved in transforming dihydroxyacetone-phosphate (DHAP) to 3-phospho glycerate (G3P) was downregulated (−1.07). In contrast to previous studies which showed a reduction in expression of genes involved in photosynthesis in response to limitation of nitrogen [15], a decrease in abundance of proteins involved in photosynthesis could not be recorded in +N P4, suggesting here a different limitation as trigger for lipid accumulation. For instance, carbon stockpiling has been identified as a response to excessive photosynthetic carbon fixation during high light conditions [39]. In conclusion, the high lipid-containing cells in this nitrate supplemented *N. oceanica* culture have geared their metabolism towards fixation of CO_2_ and lipid biosynthesis as a form of carbon stockpiling.

### −N P4 population proteome indicates cellular lipid saturation

For the −N condition of 1264 identified and quantified proteins, 156 were significantly regulated between subpopulations −N P3 and −N P4. The decrease of photosynthetic proteins, light-harvesting proteins, chlorophyll biosynthesis and proteins belonging to such complexes is a sign of decreased photosynthetic activity and electron flow leading to a reduced supply of reducing agents for biosynthesis and was also featured in a previous study about adaptation to −N [15]. Besides the decreased photosynthetic activity, the downregulation of enzymes involved in the Calvin cycle point to a lower activity of carbon fixation pathways. Downregulation of the Calvin cycle and of carbon fixation pathways was already identified as a consequence of nitrogen deprivation in diatoms [40]. For the transcriptome of *N. oceanica* comparing +N and −N with each other, 3255 differentially expressed genes were detected [15]. Major remodeling of photosynthetic activity taking place during nitrogen starvation was also reported in other studies [41]: on the one hand, chlorophyll content decreased, while the carotenoid content increased. On the other hand, the PSII photosynthetic center was downregulated during nitrogen deprivation [41]. These processes were also found for the −NP4 subpopulation indicating a more progressed adaptation to nitrogen limitation in comparison to −N P3.

Furthermore, lower abundance of the lipid droplet protein in −N P4 hints at an increase in lipid droplet size. It has been established by experiments in another study for *C. reinhardtii* that downregulation of the lipid droplet surface protein leads to larger lipid droplets which contain more lipids [42]. It may also indicate increased mobilization of stored lipids for metabolism. The downregulation of the photosynthesis and carbon fixation pathways, and upregulation of lipid degrading enzymes as lysophospholipase II, and other lipases underpin this hypothesis.

Altogether, proteome data suggest that accumulation of lipids in *N. oceanica* is not a linear process, synchronized between all population members, but that in −N P4 in contrast to −N P3, a level of lipid accumulation was reached where lipid production ceased. In cells which show a complete adaptation to the nitrate deprived state, a strong decrease in chlorophyll content was observed [41]. In this vein, analysis of the phenotypic heterogeneity in *C. cryptica* showed that lipid accumulation does not follow a linear development but shows cessations in between, where lipid metabolism is downregulated [21]. In this study, a decrease of lipid accumulation was described between 48 and 72 h during nitrogen deprivation. Also here, the occurrence of lipid-hyperaccumulated subpopulations was reported, which occurred after prolonged slow lipid accumulation. These hyperaccumulated subpopulations were stable after 2 weeks of nitrogen deprivation showing that the maximum of lipid content was reached for these cells. Arrest of lipid production could be similar to arrest of cell division and lipid accumulations, which was reported for *T. pseudonana* under silicium deprivation conditions [43].

### Recultivation experiments refute genetic heterogeneity and might follow the fractal theory

The underlying hypothesis was that if the observed population heterogeneity was based on genetic changes, the lipid accumulation features would be inherited in the sorted subpopulations This should manifest in Nile Red fluorescence intensities differing between the recultivated subpopulation +N P3 and +N P4. Instead of this, FACS results of the recultivated cells in Additional file 1: Figure S5 are very similar to original cultivation. The occurrence of +N P3 and +N P4 subpopulations in cultures grown from sorted +N P3 cells and sorted +N P4 cells has shown that under the cultivation conditions used in this study, from either starting point, i.e., P3 or P4, *N. oceanica* population would come to the same phenotypic pattern observed before sorting. This phenotype pattern proved that the population heterogeneity in the *N. oceanica* cultivation was not caused by hereditary genetic changes under our experimental conditions. Several reports documenting highly dynamic population heterogeneities in course of nitrogen starvation for microalgae as *C. reinhardtii, Euglena gracilis*, and *N. oceanica* [16, 44, 45] corroborate this conclusion. Nonetheless, genome sequence results are required to exclude genetic changes in *N. oceanica* subpopulations during +N and even −N conditions. Besides, not in all cases, where rounds of FACS selection for *N. oceanica* individuals with increased lipid content and their recultivation were done, this feature could be inherited [18]. Thus, for guiding strain breeding of lipid-accumulating *N. oceanica*, microbial population heterogeneity development and its heredity should be always investigated and ideally accompanied by OMICS analyses.

Though two subpopulations of *N. oceanica* have shown differentiations on phenotypical levels, we have noticed that after recultivation, resorting and reanalysis, both of the two sub-population have shown similar phenotypical patterns, with similar population densities and similar lipid accumulations (Additional file 1: Figure S5). This is in agreement with the fractal theory, which indirectly refutes genetic heterogeneity. Fractal theory refers to the pattern that would occur at both micro- and macro-scales [46, 47], and such recurring pattern would always reflect ecological patterns for the microbial community [48, 49].

## Conclusion

All in all, for the first time using Nile Red lipid stain, together with single-cell sorting, *N. oceanica* were distinguished into two groups for the +N and −N culture conditions. This is the first proof of population heterogeneity in *N. oceanica* using FACS. In light of previously reported population heterogeneity for several lipid-producing microalgae, we suspect this phenomenon to be widespread and relevant for possible exploitation of these organisms in an industrial setting. We have identified and confirmed the heterogeneities among *N. oceanica* populations on phenotypic and proteomic level, as well as refuting genomic heterogeneities. Furthermore, these differentiations could be recognized on population as well as on single-cell level. Although such heterogeneity patterns have been affected by growth status and lipid accumulation or saturation of the *N. oceanica* populations, pattern differentiation itself seems to be very robust against these factors. Also, we have observed that subpopulation heterogeneities might follow the fractal theory, though more time series evidences would be needed to prove this. This study demonstrates the potential of modern OMICS techniques, such as single-cell phenotyping and multiple omics data integration, to help us for better understanding of the universal patterns of microbial population heterogeneity. However, more problems emerge about this topic, e.g., robustness and plasticity of such heterogeneous pattern, the association of genotype and phenotype for such differences and their temporal–spatial dynamics. We believe that based on more OMICS and time-series data, as well as more in-depth modeling, the concerted effects that have shaped the heterogeneities among *N. oceanica* populations will be better understood. The application indications of this work are obvious: Only if heterogeneity is of genomic origin, the modern molecular breeding techniques for fast selection of superior strains of oleaginous algae are straightforward applicable. This work demonstrates the requirement of long-term continuous monitoring to clarify origin of heterogeneity.

## Additional file

**Additional file 1.** Additional Material and methods, Figures and Tables.

## Abbreviations

TAG: triacylglycerol
FACS: fluorescence-activated cell sorting
−N: nitrogen deplete
+N: nitrogen replete
−N P3: sorted subpopulation with lower Nile Red intensity of the –N *N. oceanica* cells
−N P4: sorted subpopulation with higher Nile Red intensity of the –N *N. oceanica* cells
+N P3: sorted subpopulation with lower Nile Red intensity of the +N *N. oceanica* cells
+N P4: sorted subpopulation with higher Nile Red intensity of the +N *N. oceanica* cells
TCA: tricarboxylic acid
cbbx: calvin–benson–bassham cycle-related enzyme
enoyl-ACP-reductase: enoyl-acyl carrier protein reductase
2D-PAGE: two-dimensional polyacrylamide gel electrophoresis
OD750: optical density at 750 nm
RT: room temperature
LCSM: laser scanning confocal microscopy
PPDK: pyruvate orthophosphate dikinase
RUBISCO: ribulose-1,5-bisphosphate carboxylase
TIM: triosephosphate isomerase
LACS: long-chain acyl CoA synthetase
M6PR: NADPH-dependent Mannose-6-phosphate reductase
PCA: principal component analysis
DHAP: dihydroxyacetone-phosphate
G3P: 3-phospho glycerate
MS: mass spectrometry
DMSO: dimethyl sulfoxide
DAPI: 4′,6-diamidino-2-phenylindole
BODIPY: 4,4-difluoro-1,3,5,7-tetramethyl-4-bora-3a,4a-diaza-s-indacene
FDR: false discovery rate
