# Supplementary material for: Proteomic study uncovers molecular principles of single-cell-level phenotypic heterogeneity in lipid storage of Nannochloropsis oceanica
Source: Biotechnol Biofuels. 2019 Feb 4;12:21. doi: 10.1186/s13068-019-1361-7 (PMC6360718; doi:10.1186/s13068-019-1361-7)
Supplement: Supplementary file 1 — Additional file 1. Additional Material and methods, Figures and Tables. [file 13068_2019_1361_MOESM1_ESM.docx]

**Additional Information**

**Proteomic study uncovers molecular principles of single-cell level phenotypic heterogeneity in lipid storage of *Nannochloropsis oceanica***

Chaoyun Chen^1,┼^, Andreas Harst^2,┼^, Wuxin You^2,4^, Jian Xu^4^, Kang Ning^1,*^, Ansgar Poetsch^2,3*^

*^1^ Key Laboratory of Molecular Biophysics of the Ministry of Education, Hubei Key Laboratory of Bioinformatics and Molecular-imaging, Department of Bioinformatics and Systems Biology, College of Life Science and Technology, Huazhong University of Science and Technology, Wuhan, Hubei 430074, China*

*^2^ Plant Biochemistry, Ruhr University Bochum, 44801 Bochum, Germany*

^3^ *School of Biomedical and Healthcare Sciences, Plymouth University, Plymouth PL4 8AA, UK*

^4^*Single-Cell Center, CAS Key Laboratory of Biofuels and Shandong Key Laboratory of Energy Genetics, Qingdao Institute of BioEnergy and Bioprocess Technology, Chinese Academy of Sciences, Qingdao, Shandong, 266101, China*

^┼^These authors contributed equally to this work.

^*^Corresponding authors. E-mail: [ansgar.poetsch@ruhr-uni-bochum.de](mailto:ansgar.poetsch@ruhr-uni-bochum.de), ningkang@hust.edu.cn

**Content**

**Additional Materials & Methods 3**

Fluorescence microscopy of *N. oceanica* 3

Emission and Excitation Fluorescence Spectrophotometry 3

Cell counting and lipid content comparison 4

**Additional Figures 5**

Figure S1. Comparison of Nile Red staining of +N and –N *N. oceanica* cells 5

Figure S2. Plot of SSC vs FSC and FCS vs Nile Red fluorescence 6

Figure S3. Fluorescence microscopy analysis of stained +N and –N N. oceanica cells 7

Figure S4. Proteomic differences of the of subpopulations 9

Figure S5. SSC vs Nile Red intensity of +N and –N cultivation cells in FACS 10

Figure S6. Population heterogeneity in the Recultivation of sorted +N subpopulations 11

Figure S7. Population heterogeneity of the cultivation in the small scale photobioreactor 12

**Additional Tables 14**

Table S1. Protein quantification results of +N P4 and +N P3 14

Table S2. Protein quantification results of –N P4 and –N P3 16

Table S3. Protein quantification results of +N and –N 21

**Reference 33**

# Additional Materials & Methods

## Fluorescence microscopy of *N. oceanica*

Nile Red stain [1]: for staining with Nile Red, *N.oceanica* cells were aliquoted in the required number as determined by cell counting. The aliquoted cells were washed three times using -N f/2 medium (12000 g, 2 min, Eppendorf 5804, at RT). Cells were resuspended in 1 mL mixture of -N f/2 medium + 5% DMSO. Nile Red solution (10µl) was added to sample (1 mL) from a 0.1 g/L Nile Red stock in acetone solution. The samples were quickly vortexed and then stored in the dark for at least 6 minutes. The stained sample was then washed twice with sterile milliQ water to remove any dye still present in solution, then resuspended in 0.5 mL sterile milliQ water.

Bodipy stain: neutral lipids were imaged in *N. oceanica* by staining with BODIPY 505/515 (4,4-difluoro-1,3,5,7-tetra-methyl-4-bora-3a,4adiaza- s-indacene) according to the protocol outlined by Rumin et al[2] with minor modifications. Briefly, 1 mL of culture was centrifuged at 12,000 ×g for 2 min and the pellet was washed three times with -N f/2 medium. Cells were then resuspended in 1 mL glycerol (0.10g mL−1) supplemented with BODIPY 505/515 (final concentration of 1 μg mL−1), and vortexed for 1 min before being incubated in the dark for 5 min at room temperature. The stained sample was then washed twice with sterile milliQ water to remove any dye still present in solution, then resuspended in 0.1 mL sterile milliQ water.

LCSM: Stained cells from both +N and -N treatments were imaged using a laser confocal scanning microscope (100x objective oil lens 10x ocular lens, Olympus FV1000) to quantify lipid droplets in the algal cells. Inmate stack was generated using FV10-ASW Viewer version 4.2. Chlorophyll fluorescence was observed using a Cy-5 filter (excitation 633 nm and emission 661-761 nm) and lipid fluorescence was observed using a FITC filter (excitation 488 nm and emission 500-550 nm) for BODIPY and same filter with excitation 488 nm and emission 500-600nm for Nile Red. Offset was set to 100% to remove the background noise. To display results, channel 1 from the fluorescence dye was set to red and channel 2 from chloroplast autofluorescence was set to green.

## Emission and Excitation Fluorescence Spectrophotometry

Fluorescence spectrophotometry experiments were performed with +N and -N *N. oceanica* cultures grown as described in **Materials and Methods** sections, then the aliquots were diluted to a cell concentration of 2 million cells per mL. The cells were stained according to the Nile Red staining protocol described in **Materials and Methods**, but diluted to 2 million cells per mL. 1 mL of stained cells was pipetted into plastic fluorescence cuvettes for measurement.

Fluorescence spectrophotometric measurements were performed with a model FP-6500 Spectrofluorometer from Jasco. Spectrum measurement mode was set in the parameter box of the Spectra Manager software. Wavelength of excitation was set to 480 nm with an excitation bandwidth of 1 nm. Parameters for emission spectra were emission bandwidth of 1 nm, scanned wavelength ranges were decided for emission spectra; the ranges lay between 480 nm and 660 nm. Response time was set to two seconds, sensitivity was set to manual, scanning speed was set to 200 nm/min and PMT voltage was set to 300 V. All measurements were performed against a blank containing only 35 g/l sea salt water and 15 % DMSO.

**Cell counting and lipid content comparison**

Cell number was counted with MoFlo™ XDP High speed cell sorter, reported with the Summit software V5.5.0.1688. Detected by MoFlo™ XDP High speed cell sorter, the averaged Nile Red intensity of each subpopulation was calculated with Summit. The comparison of lipid content of –N and +N cultivation was performed with Nile Red intensity of subpopulations. Calculation of the relative lipid content ratio of -N to +N cultivation: Firstly, the absolute abundance of each subpopulation was multiplied with the averaged Nile Red intensity for the relative Nile Red intensity of each subpopulation; Secondly, the relative Nile Red intensity of the two subpopulations of each culture condition was summed up to obtain the relative Nile Red intensity of the +N and -N cultivation; Then, the relative Nile Red Intensity of –N was divided by the Nile Red Intensity of +N for the lipid content ratio.

## Additional Figures

**
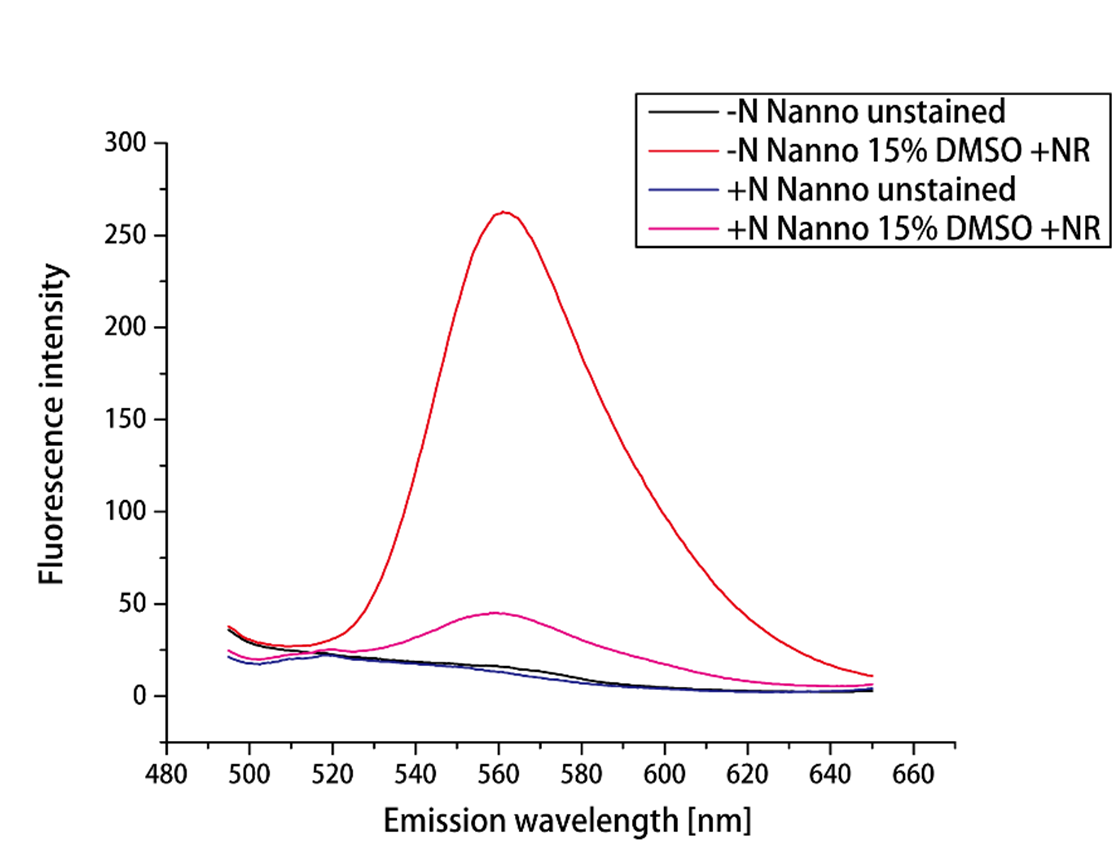
**

**Figure S1. Fluorescence emission spectra of *N. oceanica* cultivated in –N or +N medium.** Cells grown under –N and +N conditions were stained with Nile Red in presence of 15% DMSO. Additionally, controls were performed for both conditions without Nile Red and DMSO. Cells were excited using light of 480nm wavelength

**
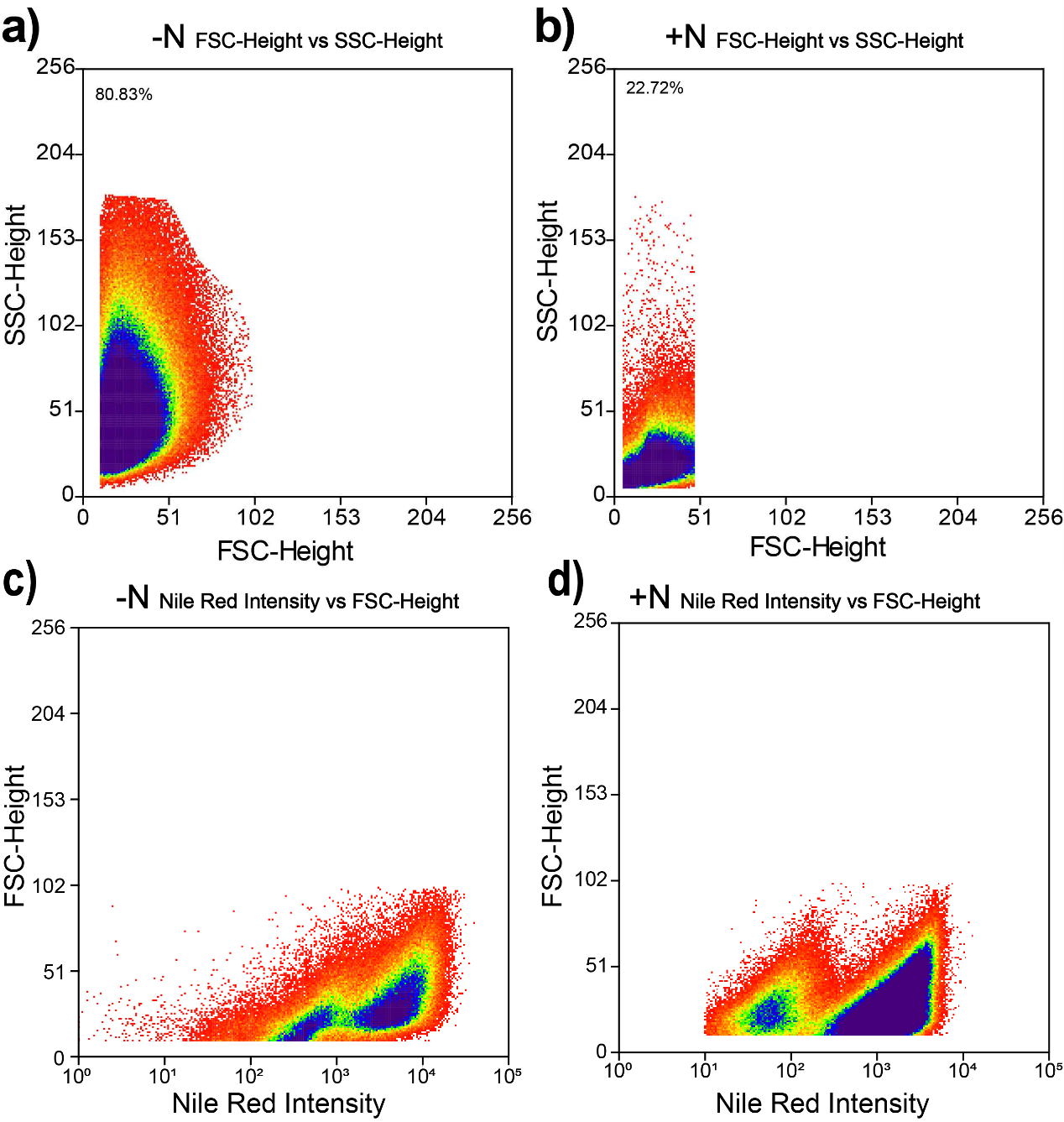
**

**Figure S2: Plot of SSC vs FSC and FCS vs Nile Red fluorescence.** Detected by MoFlo™ XDP High speed cell sorter, a) shows the SCC-height and FSC-height histogram of +N cultivation sample, and the input sample was the same as in **Figure 2 b)**; while b) presents SCC-height and FSC-height histogram of -N cultivation sample, and the input sample was the same as in **Figure 3 a)**. Comparison between FSC vs Nile Red and SSC vs Nile Red of the + N cultivation sample; c) shows -N, d) shows the +N.

1. Comparison of Nile Red and BODIPY stain


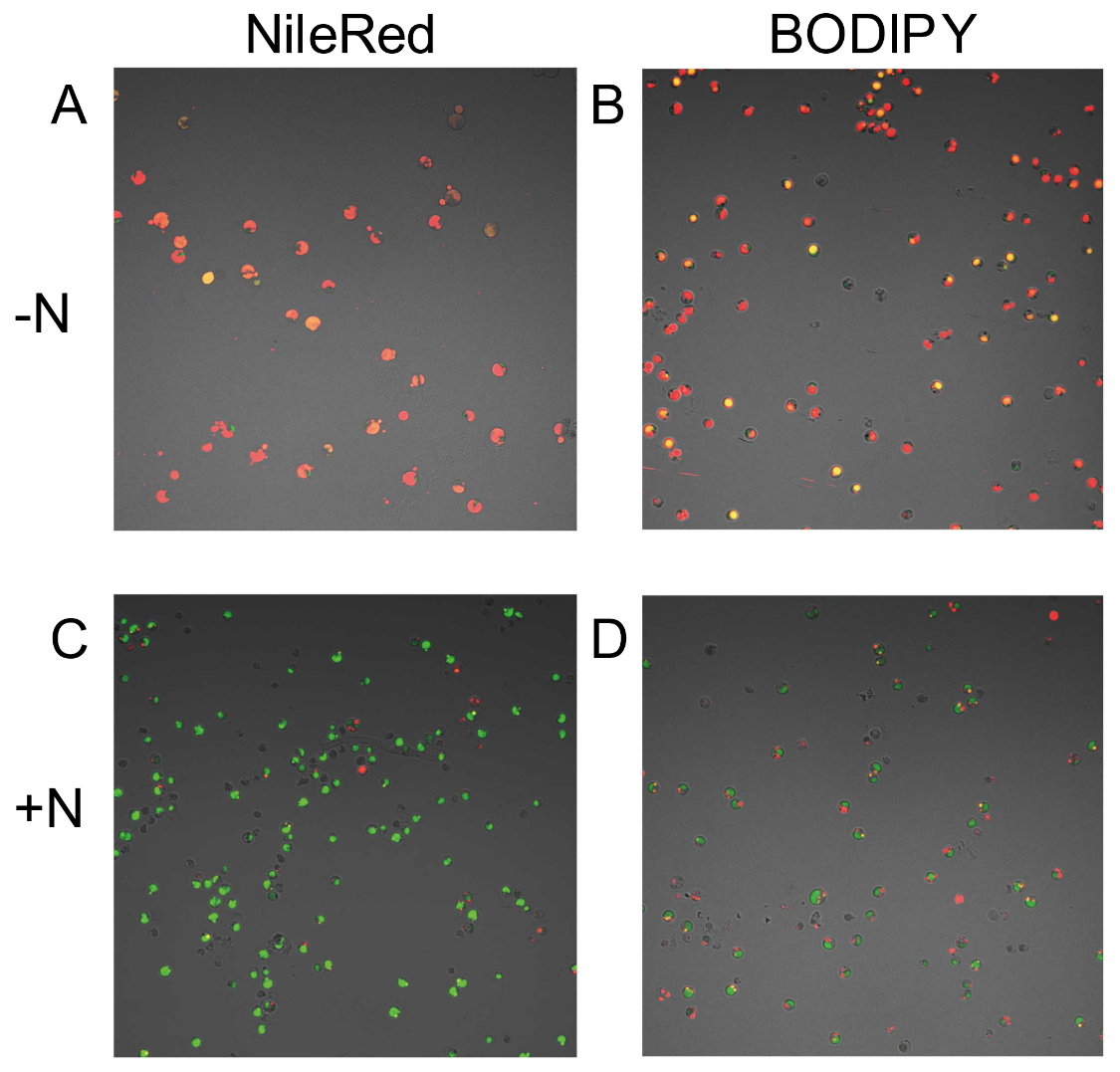


1. Single cell image of *N. oceanica* stained with BODIPY


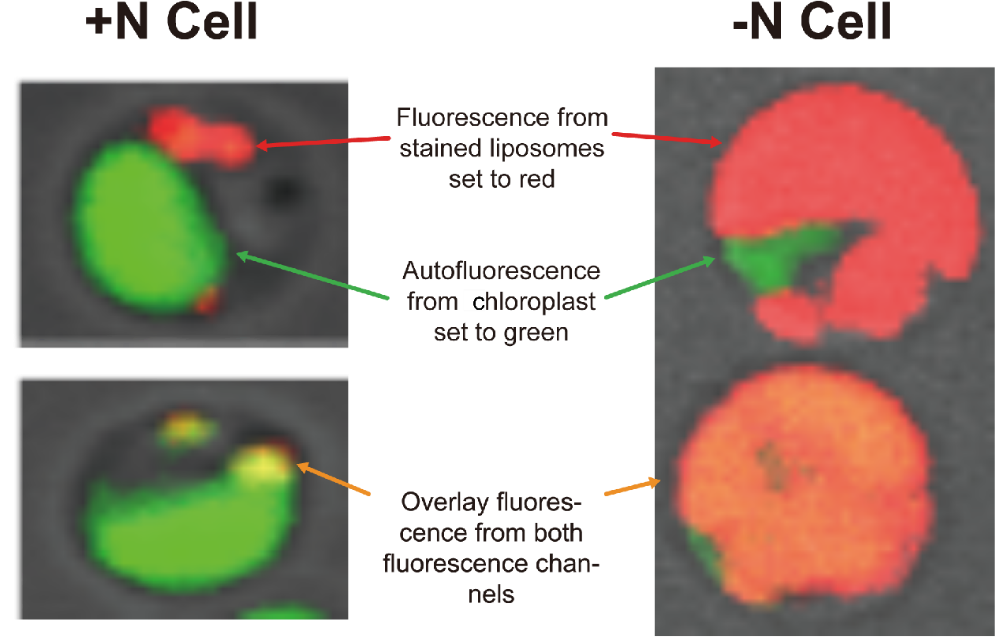


1. Phenotypic heterogeneity for +N and –N cells


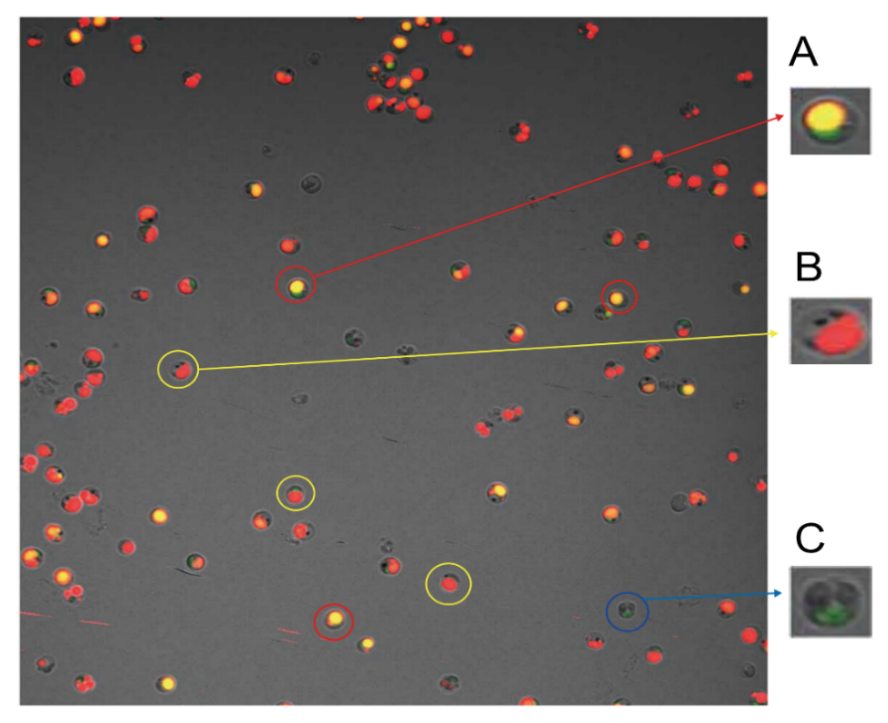

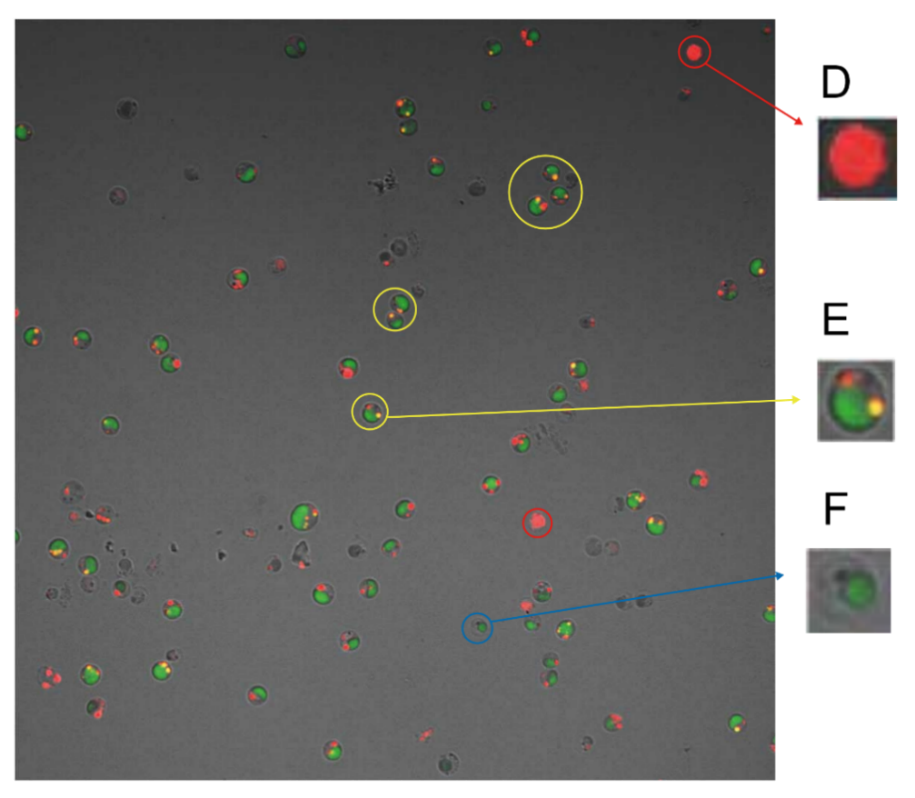


-N Cells

+N Cells

**Figure S3.** **Fluorescence microscopy analysis of stained +N and –N *N. oceanica* cells.** **a)** Fluorescence micrograph of *N. oceanica* cells in +N and –N (+/- nitrate) conditions stained with Nile Red or BODIPY (red channel) and detection of chlorophyll autofluorescence (green channel). **b)** Single cell image displaying stained structures using BODIPY stain. **c)** BODIPY-stained +N and -N cells, selected single cell pictures: A both liposome and unfolded chloroplast, B big liposome, no chloroplast, C chloroplast, but no liposome, D big liposome, no chloroplast, E normal-sized chloroplast, small liposomes, F chloroplast, but no liposome.


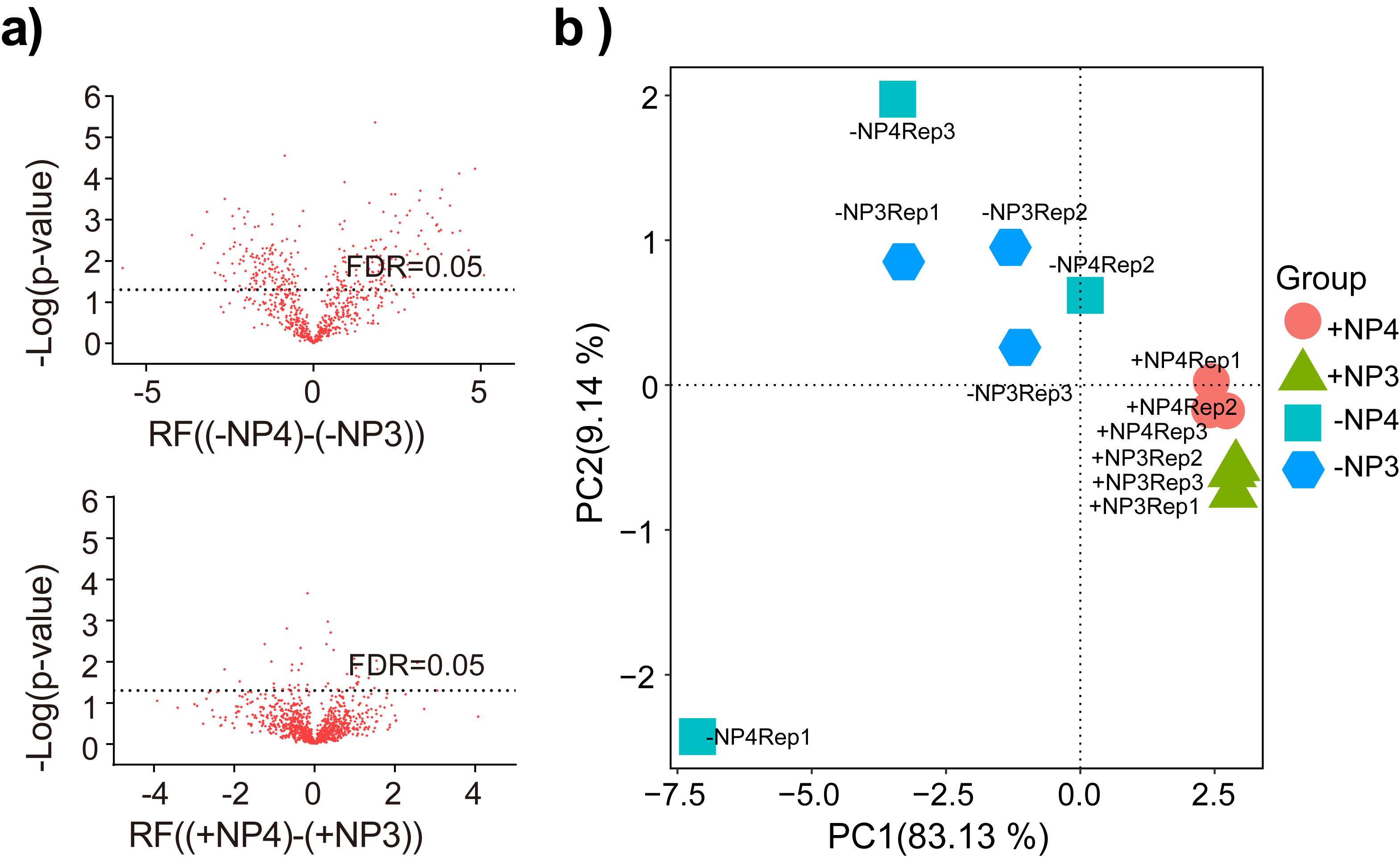
**Figure S4. Proteomic differences of the subpopulations. a)** Volcano plots of the subpopulations sorted from the -N *N.oceanica* cultures (upper panel) and sorted from the +N *N. oceanica* cultures (lower panel), p-values were calculated with a two sample t-test. Regulation factors were calculated by subtracting Log2 median-normalized abundance values of -N P3 and +N P3 values from -N P4 and +N P4 values. The FDR value line shown in the figures equals to an FDR of 0.05 calculated by Perseus. **b)** PCA comparing the replicates of +N P3 and +N P4 subpopulations with the replicates of the -N P3 and –N P4 subpopulations. The first principal component has a loading of 83.13%, while the second principal component has a loading of 9.14%.


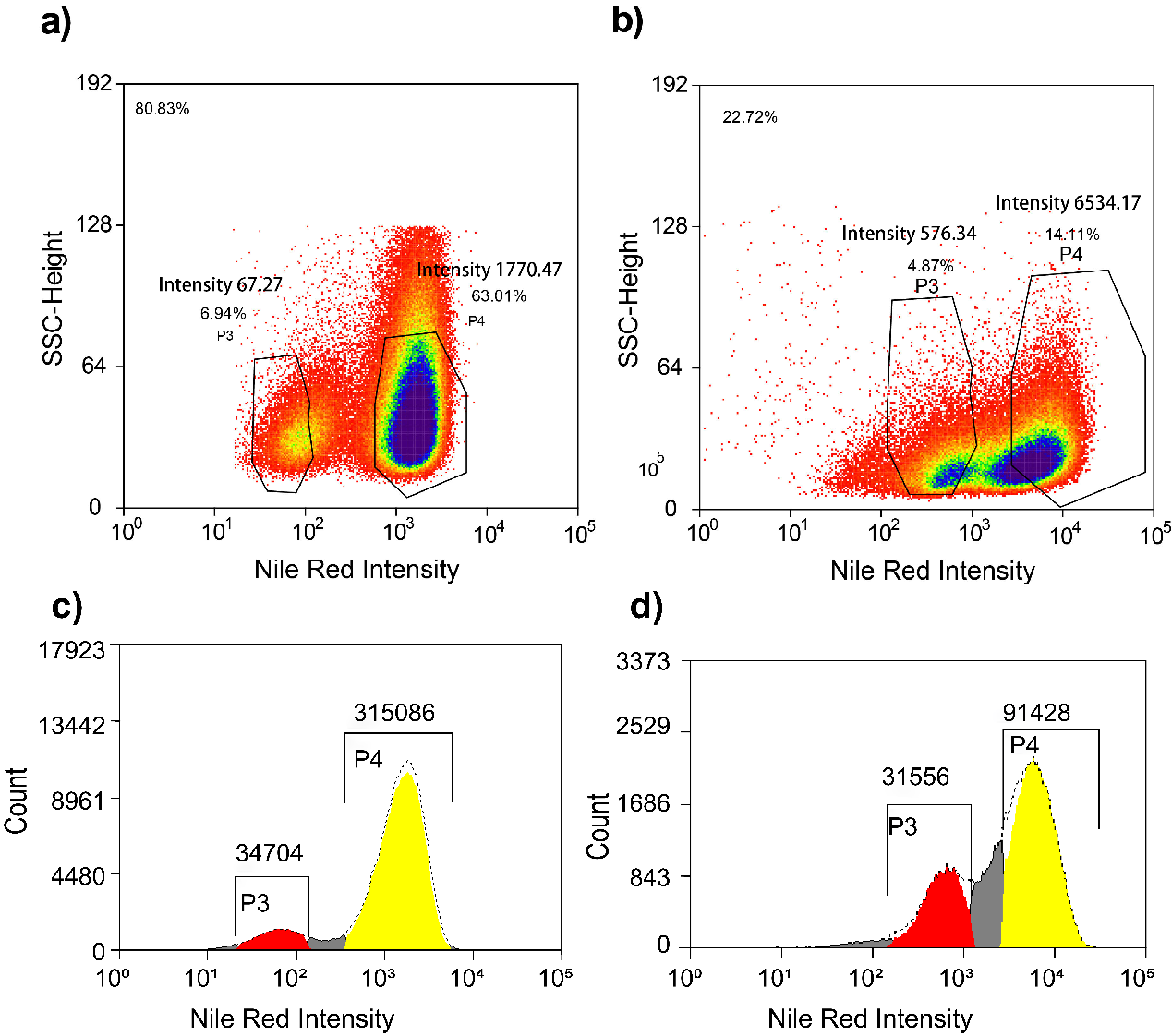


**Figure S5. SSC vs Nile Red intensity of +N and –N cultivation cells in FACS.** **a)** Cell density plot +N *N. oceanica* cells stained with Nile Red, the SSC (Sidescatter)-height channel informs about morphological features, while the Nile Red intensity axis value reports the intensity of Nile Red fluorescence, **b)** Cell density plot -N *N. oceanica* cells stained with Nile Red, the SSC (Sidescatter) height channel informs about morphology, while the FL2-Log-height channel reports the intensity of the Nile Red fluorescence. In this case two subpopulations exist with one showing increased fluorescence intensity in the Nile Red. Percentages denote gated cell fraction from total. The averaged intensity of the subpopulations is indicated as ‘Intensity’ in the figure. Cell count and distribution plot of +N *N. oceanica* cells **c)** and -N *N. oceanica* cells **d)**. The Count axis informs about the cell number, while the Nile Red intensity axis value reports the intensity of Nile Red fluorescence. The number above bar represents the total cell number in the gate, and color red corresponds to subpopulation P3 with lower Nile Red intensity, color yellow corresponds to subpopulation P4 with higher Nile Red intensity.


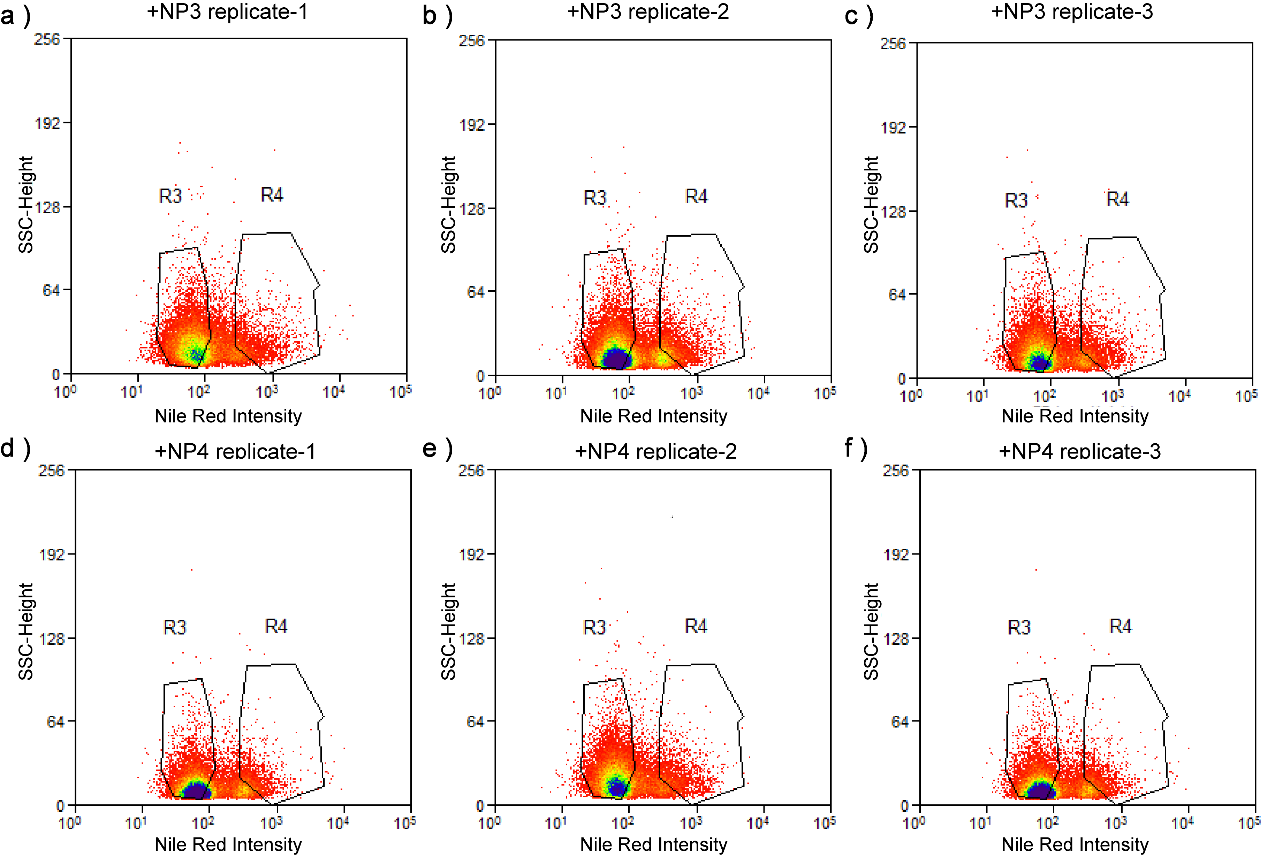
**Figure S6. Population heterogeneity in the recultivation of sorted +N subpopulations.** +N P3 and +N P4 subpopulations were sorted from a +N *N. oceanica* culture onto filters for recultivation. Cells from these filters were then used to inoculate freshly prepared +N medium separately with +N P3 or +N P4 in three replicates each. After sufficient growth of the subpopulation cultures, samples from these cultures were stained with Nile Red and analyzed with FACS. **a)**, **b)** and **c)** show sorting of recultivation for the +N P3 subpopulation and **d)**, **e)** and **f)** of recultivation for the +N P4 subpopulation.


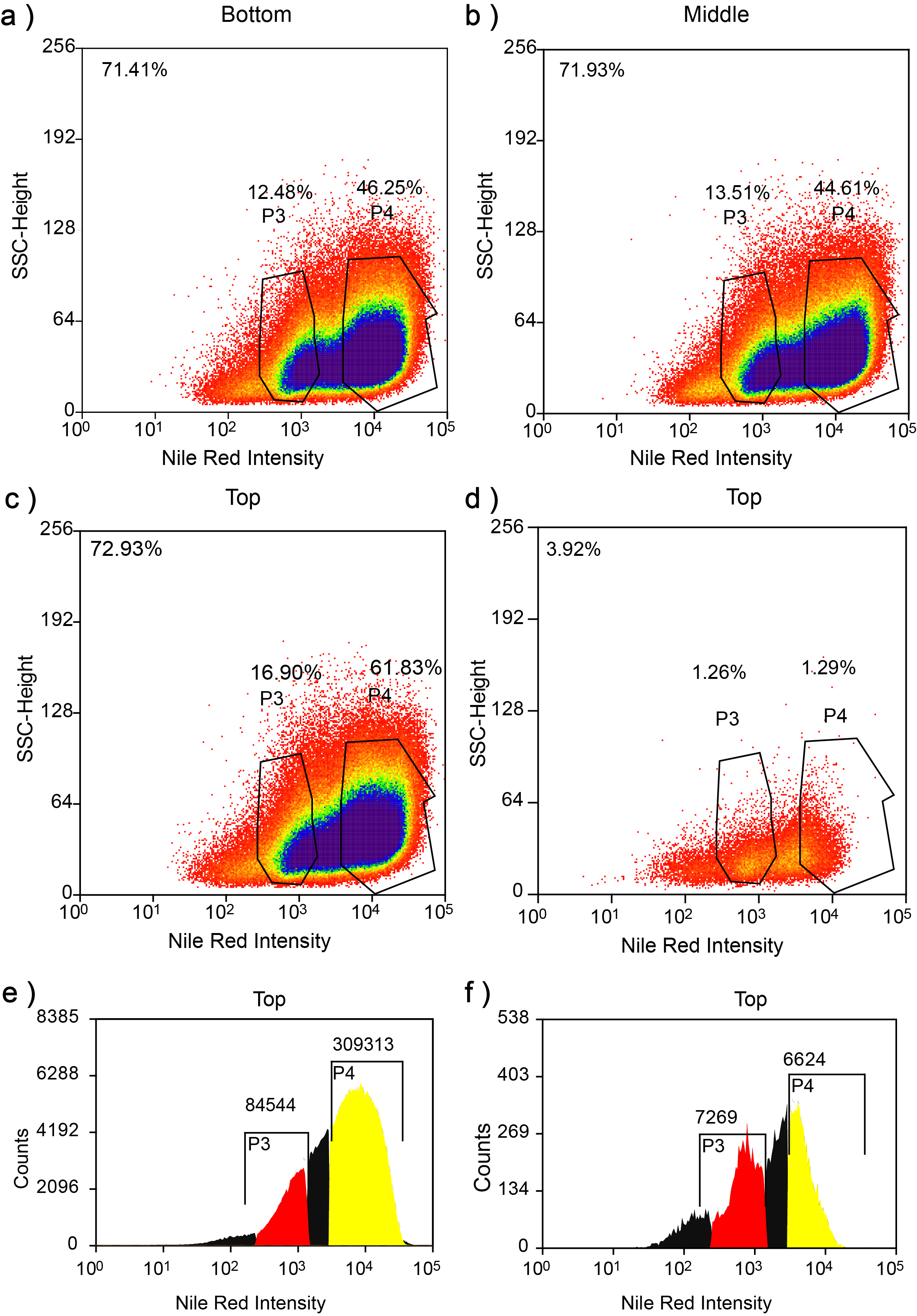
 **Figure S7. Population heterogeneity of the cultivation in the small scale photo bioreactor**. **a)**, **b)**, **c)** represent the FACS analysis results of the samples collected from the bottom, middle, and top part of the bioreactor respectively. The signal distribution patterns are the same in the **a)**, **b)**, **c)**. **d)** same data as in **c)**, i.e. cells from top of bioreactor, but only 3.92% of total signals input is shown to facilitate visual discrimination of the subpopulations. **e)** cell count and distribution plot of **c)**. The Count axis informs about the cell number, while the Nile Red intensity axis value reports the intensity of Nile Red fluorescence. The number above bar represents the total cell number in the gate, and color red corresponds to subpopulation P3 with lower Nile Red Intensity, color yellow corresponds to subpopulation P4 with higher Nile Red Intensity. **f)** is the cell count and distribution plot of **d)**.

# Additional Tables

**Table S1. Protein quantification results of +N P3 and +N P4.** Proteins showing significant regulation between subpopulation +N P3 and subpopulation +N P4 based on a two-sample-t-test. Proteins were identified as being significantly regulated between +N P3 and +N P4 with a FDR value below 0.05. The regulation factor (RF) was calculated by subtracting log2 median normalized quantification values +N P3 from +N P4.

| **Gene ID** | **Description** | **p-value between subpopulations** | **RF (P4-P3)** |
| --- | --- | --- | --- |
| ***IMET 29*** | PREDICTED PROTEIN | 2.16E-04 | -0.17 |
| ***IMET 4963*** | RNA HELICASE REQUIRED FOR BINDING mRNA TO RIBOSOME | 1.06E-03 | 0.33 |
| ***IMET 4437*** | PREPROTEIN TRANSLOCASE SUBUNIT SecA | 1.55E-03 | -0.69 |
| ***IMET 920*** | HYPOTHETICAL PROTEIN | 1.95E-03 | 0.41 |
| ***IMET 8920*** | PREDICTED PROTEIN | 3.75E-03 | 0.30 |
| ***IMET 1872*** | PRE-mRNA-SPLICINGFACTOR CDC5/CEF1 | 3.76E-03 | -1.24 |
| ***IMET 8004*** | HEAT SHOCK PROTEIN | 4.63E-03 | -0.35 |
| ***IMET 8757*** | PLASTID FtsZ INVOLVED IN CELL DIVISION | 5.14E-03 | 0.47 |
| ***IMET 5268*** | 26S PROTEASOME REGULATORY SUBUNIT | 8.39E-03 | 0.99 |
| ***IMET 5133*** | PYRUVATE,PHOSPHATE DIKINASE | 9.44E-03 | 1.54 |
| ***IMET 3984*** | TRIOSEPHOSPHATE ISOMERASE | 9.84E-03 | -1.07 |
| ***IMET 433*** | PEPTIDYL-PROLYL CIS-TRANS ISOMERASE | 1.06E-02 | 2.56 |
| ***IMET 9275*** | TonB-DEPENDENT RECEPTOR | 1.12E-02 | -0.32 |
| ***IMET 4270*** | OXIDOREDUCTASEDOMAIN PROTEIN | 1.18E-02 | -0.56 |
| ***IMET 985*** | CYSPEROXIREDOXIN | 1.41E-02 | 1.03 |
| ***IMET 8424*** | RIBOSOMAL PROTEIN RPS15 | 1.52E-02 | 1.56 |
| ***IMET 9117*** | HALOACIDDEHALOGENASE-LIKE HYDROLASE PROTEIN | 1.54E-02 | -2.24 |
| ***IMET 4156*** | ARGONAUTE 1 | 1.58E-02 | -0.39 |
| ***IMET 7611*** | ASPARTYL BETA-HYDROXYLASE | 1.63E-02 | -0.57 |
| ***IMET 1767*** | PREDICTED PROTEIN | 1.99E-02 | 0.81 |
| ***IMET 8732*** | ENHANCER OF RUDIMENTARY HOMOLOG | 2.19E-02 | 1.04 |
| ***IMET 4666*** | LIPIDDROPLET SURFACE PROTEIN | 2.33E-02 | 1.10 |
| ***IMET 516*** | PREDICTED PROTEIN | 2.50E-02 | 1.35 |
| ***IMET 665*** | NADPH DEPENDENT MANNOSE 6-PHOSPHATE REDUCTASE | 2.99E-02 | -1.87 |
| ***IMET 2490*** | T-COMPLEX PROTEIN 1 SUBUNIT EPSILON (CHAPERONE) | 3.09E-02 | 1.08 |
| ***IMET 6998*** | PREDICTED PROTEIN | 3.29E-02 | 1.05 |
| ***IMET 3775*** | D-3-PHOSPHOGLYCERATEDEHYDROGENASE | 3.36E-02 | 0.34 |
| ***IMET 6758*** | ADENOSINE KINASE | 3.39E-02 | -1.02 |
| ***IMET 1832*** | CbbX PROTEIN HOMOLOG (ACTIVATOR OF RUBISCO) | 3.57E-02 | 1.07 |
| ***IMET 6219*** | CHAPERONIN | 3.63E-02 | -0.18 |
| ***IMET 4984*** | 3-ISOPROPYLMALATE DEHYDROGENASE | 3.68E-02 | -0.40 |
| ***IMET 4807*** | VACUOLAR SORTING PROTEIN | 3.73E-02 | -0.62 |
| ***IMET 5201*** | AP-1 COMPLEX SUBUNIT BETA-1 | 4.01E-02 | 0.28 |
| ***IMET 1991*** | HYPOTHETICAL PROTEIN | 4.23E-02 | -0.49 |
| ***IMET 7472*** | CATION-TRANSPORTING ATPASE | 4.24E-02 | -1.02 |
| ***IMET 7085*** | HYPOTHETICAL PROTEIN | 4.27E-02 | 0.89 |
| ***IMET 9488*** | V-TYPE PROTON ATPASE SUBUNIT F | 4.31E-02 | 0.97 |
| ***IMET 7822*** | MPV17-LIKE PROTEIN | 4.37E-02 | 1.49 |
| ***IMET 1484*** | FUCOXANTHIN CHLOROPHYLL PROTEIN | 4.57E-02 | -0.21 |
| ***IMET 3596*** | LONG CHAIN ACYL-CoA SYNTHETASE | 4.64E-02 | -0.79 |
| ***IMET 3478*** | HOMOSERINE DEHYDROGENASE | 4.67E-02 | 0.58 |
| **IMET 7974** | HYPOTHETICAL PROTEIN | 4.70E-02 | -0.64 |

**Table S2. Protein quantification results of -N P3 and -N P4.** Proteins showing significant regulation between subpopulation -N P3 and subpopulation -N P4 based on a two-sample-t-test. Proteins were identified as being significantly regulated between -N P3 and -N P4 with a p-value below 0.05. The regulation factor (RF) was calculated by subtracting log2 median normalized quantification values of -N P3 from -N P4.

| **Gene ID** | **Description** | **p-value between subpopulations** | **RF (P4-P3)** |
| --- | --- | --- | --- |
| ***MET 7335*** | ACETYLORNITHINE DEACETYLASE | 4.33E-06 | 1.85 |
| ***IMET 8782*** | MPV17-LIKE PROTEIN | 2.79E-05 | -0.86 |
| ***IMET 6737*** | PEPTIDYLPROLYL ISOMERASE | 5.76E-05 | 4.83 |
| ***IMET 8124*** | THIOREDOXIN-1 | 7.52E-05 | 4.35 |
| ***IMET 2756*** | TB2/DP1/HVA22-RELATED PROTEIN | 1.22E-04 | 0.93 |
| ***IMET 1036*** | EPIMERASE 4-REDUCTASE | 1.85E-04 | 3.84 |
| ***IMET 3060*** | TRANSITIONAL ENDOPLASMIC RETICULUM ATPASE | 1.98E-04 | 3.20 |
| ***IMET 74*** | UNCHARACTERIZED PROTEIN | 2.39E-04 | 2.33 |
| ***IMET 1149*** | UNCHARACTERIZED PROTEIN | 2.41E-04 | 2.44 |
| ***IMET 6784*** | UNCHARACTERIZED PROTEIN | 3.00E-04 | 3.79 |
| ***IMET 4444*** | GERANYLGERANYL REDUCTASE | 3.12E-04 | -2.65 |
| ***IMET 8373*** | UNCHARACTERIZED PROTEIN | 3.37E-04 | 3.16 |
| ***IMET 9888*** | NADH-UBIQUINONE OXIDOREDUCTASE CHAIN 5 | 3.89E-04 | 1.67 |
| ***IMET 7843*** | CUPIN 4 FAMILY PROTEIN | 4.54E-04 | 4.08 |
| ***IMET 2919*** | 30S RIBOSOMAL PROTEIN S15 | 5.38E-04 | -2.22 |
| ***IMET 6414*** | CARBOXYL-TERMINAL PROTEASE | 6.08E-04 | 2.88 |
| ***IMET 7547*** | CHAPERONIN CPN60/TCP-1 | 6.14E-04 | -0.31 |
| ***IMET 5459*** | RIBOSOMAL PROTEIN S25 | 6.38E-04 | -1.97 |
| ***IMET 5915*** | HEAT SHOCK PROTEIN HSP20 | 6.40E-04 | 2.09 |
| ***IMET 9873*** | PHOTOSYSTEM I REACTION CENTER SUBUNIT XI | 6.42E-04 | -3.18 |
| ***IMET 4031*** | BETA-LACTAMASE/TRANSPEPTIDASE-LIKE PROTEIN | 7.07E-04 | 3.41 |
| ***IMET 4634*** | RIBULOSE-PHOSPHATE 3-EPIMERASE | 7.39E-04 | -1.22 |
| ***IMET 1545*** | D-LACTATE DEHYDROGENASE | 7.79E-04 | 2.80 |
| ***IMET 9127*** | UNCHARACTERIZED PROTEIN | 8.09E-04 | 2.45 |
| ***IMET 473*** | UNCHARACTERIZED PROTEIN | 8.19E-04 | -2.42 |
| ***IMET 5303*** | LIGHT HARVESTING COMPLEX PROTEIN | 8.76E-04 | -2.07 |
| ***IMET 8106*** | S-ADENOSYLMETHIONINE MITOCHONDRIAL CARRIER PROTEIN | 8.90E-04 | 3.56 |
| ***IMET 5452*** | SUCCINATE--COA LIGASE [ADP-FORMING] SUBUNIT BETA, MITOCHONDRIAL | 1.07E-03 | 0.93 |
| ***IMET 7624*** | TUBULIN BETA CHAIN | 1.18E-03 | 2.58 |
| ***IMET 4803*** | UNCHARACTERIZED PROTEIN | 1.26E-03 | -2.04 |
| ***IMET 3958*** | ADENYLOSUCCINATE SYNTHETASE (PHAEODACTYLUM TRICORNUTUM) | 1.29E-03 | -1.24 |
| ***IMET 6264*** | DEAD-BOX ATP-DEPENDENT RNA HELICASE | 1.29E-03 | 0.79 |
| ***IMET 3062*** | DEHYDROGENASE | 1.30E-03 | 3.69 |
| ***IMET 6181*** | MANGANESE LIPOXYGENASE | 1.37E-03 | 3.67 |
| ***IMET 9762*** | 50S RIBOSOMAL PROTEIN L11, CHLOROPLASTIC | 1.43E-03 | -0.42 |
| ***IMET 7925*** | HISTONE FAMILY PROTEIN DNA-BINDING PROTEIN | 1.44E-03 | -1.64 |
| ***IMET 7025*** | ACETYL-COACARBOXYLASE, PARTIAL [ECTOCARPUS SILICULOSUS] | 1.50E-03 | -1.75 |
| ***IMET 1151*** | VACUOLAR (H+)-ATPASE G SUBUNIT | 1.64E-03 | -2.37 |
| ***IMET 7333*** | UNCHARACTERIZED PROTEIN | 1.68E-03 | 0.87 |
| ***IMET 1840*** | POLYKETIDE SYNTHASE | 1.77E-03 | 3.81 |
| ***IMET 2732*** | HISTONE DEACETYLASE | 1.84E-03 | 3.23 |
| ***IMET 4901*** | HYPOTHETICAL PROTEIN | 1.85E-03 | 4.38 |
| ***IMET 5348*** | PUTATIVE LIPASE | 1.90E-03 | 3.75 |
| ***IMET 1462*** | HYDROLASE | 1.91E-03 | 2.32 |
| ***IMET 9613*** | WOS2 PROTEIN | 2.06E-03 | 2.53 |
| ***IMET 3662*** | DYNAMIN-LIKE PROTEIN | 2.07E-03 | 4.16 |
| ***IMET 8873*** | CYTOCHROME B6-F COMPLEX IRON-SULFUR SUBUNIT | 2.36E-03 | -3.63 |
| ***IMET 846*** | UNCHARACTERIZED PROTEIN | 2.44E-03 | 3.14 |
| ***IMET 5304*** | LIGHT-HARVESTING PROTEIN | 2.95E-03 | -0.79 |
| ***IMET 8661*** | NAD-DEPENDENT EPIMERASE DEHYDRATASE | 3.03E-03 | 2.01 |
| ***IMET 4633*** | LIGHT-HARVESTING PROTEIN | 3.06E-03 | -1.84 |
| ***IMET 1227*** | UNCHARACTERIZED PROTEIN | 3.14E-03 | 1.87 |
| ***IMET 2436*** | GLUTAMATE DEHYDROGENASE | 3.24E-03 | -0.55 |
| ***IMET 8004*** | CLPB CHAPERONE, HSP100 FAMILY | 3.26E-03 | 1.77 |
| ***IMET 9882*** | NADH-DEHYDROGENASE SUBUNIT 7 | 3.28E-03 | -0.93 |
| ***IMET 4666*** | LIPID DROPLET SURFACE PROTEIN | 3.57E-03 | -2.35 |
| ***IMET 7298*** | RIBOSOMAL PROTEIN L15 | 3.85E-03 | -3.28 |
| ***IMET 8328*** | PEPTIDOGLYCAN-BINDING LYSIN DOMAIN CONTAINING PROTEIN | 3.86E-03 | 3.33 |
| ***IMET 9836*** | ATP SYNTHASE CF0 B CHAIN SUBUNIT I | 4.02E-03 | -2.19 |
| ***IMET 5791*** | MEMBRANE-ASSOCIATED 30KDA PROTEIN | 4.05E-03 | -1.21 |
| ***IMET 2262*** | UNCHARACTERIZED PROTEIN | 4.21E-03 | -1.23 |
| ***IMET 9470*** | HISTONE H4 | 4.35E-03 | 1.19 |
| ***IMET 7004*** | COB(I)YRINIC ACID A,C-DIAMIDE ADENOSYLTRANSFERASE, MITOCHONDRIAL | 4.47E-03 | 2.46 |
| ***IMET 6011*** | ZETA-CAROTENE DESATURASE | 4.50E-03 | -2.38 |
| ***IMET 2808*** | LYSOPHOSPHOLIPASE II | 4.51E-03 | 1.72 |
| ***IMET 9241*** | GLYOXALASE DOMAIN-CONTAINING 4-LIKE PROTEIN | 4.71E-03 | 2.17 |
| ***IMET 2789*** | H+-TRANSLOCATING PYROPHOSPHATASE FAMILY | 4.87E-03 | -3.36 |
| ***IMET 8369*** | Cu/Zn SUPEROXIDE DISMUTASE | 4.90E-03 | 1.56 |
| ***IMET 9878*** | ATP-DEPENDENT CLP PROTEASE ATPASE | 4.92E-03 | -1.39 |
| ***IMET 3052*** | THIOREDOXIN FAMILY PROTEIN | 4.94E-03 | -1.28 |
| ***IMET 6337*** | SIMILAR TO DEK ONCOGENE (DNA BINDING)(ECTOCARPUS SILICULOSUS) | 5.00E-03 | -1.52 |
| ***IMET 7302*** | UNCHARACTERIZED PROTEIN | 5.02E-03 | -2.00 |
| ***IMET 5882*** | UNCHARACTERISTIC PROTEIN | 5.09E-03 | -2.49 |
| ***IMET 6398*** | ORNITHINE AMINOTRANSFERASE | 5.09E-03 | 3.43 |
| ***IMET 4425*** | PEPTIDYL-PROLYL CIS-TRANS ISOMERASE | 5.16E-03 | 2.12 |
| ***IMET 588*** | PLASMA MEMBRANE INTRINSIC PROTEIN | 5.18E-03 | 0.78 |
| ***IMET 9804*** | PHOTOSYSTEM II D2 PROTEIN | 5.35E-03 | -1.13 |
| ***IMET 4265*** | SPLICING FACTOR 3A | 5.42E-03 | 1.80 |
| ***IMET 9*** | HEAT SHOCK PROTEIN HSP20 | 5.47E-03 | 4.64 |
| ***IMET 8619*** | UNCHARACTERIZED PROTEIN | 5.55E-03 | -1.29 |
| ***IMET 9194*** | 40S RIBOSOMAL PROTEIN S4 | 5.73E-03 | 0.92 |
| ***IMET 3007*** | PEROXIREDOXIN-LIKE PROTEIN | 5.78E-03 | 2.89 |
| ***IMET 9851*** | CYTOCHROME F | 5.83E-03 | -2.00 |
| ***IMET 9877*** | 50S RIBOSOMAL PROTEIN L19 | 6.25E-03 | -0.46 |
| ***IMET 9096*** | LIGHT-HARVESTING PROTEIN | 6.32E-03 | -1.23 |
| ***IMET 2084*** | PUTATIVE MEMBRANE PROTEIN (ANAPLASMA PHAGOCYTOPHILUM ST.) | 6.46E-03 | -0.95 |
| ***IMET 6283*** | ATP SYNTHASE SUBUNIT DELTA | 6.54E-03 | -0.67 |
| ***IMET 4284*** | UNCHARACTERIZED PROTEIN | 6.58E-03 | -1.46 |
| ***IMET 7250*** | HEAT SHOCK PROTEIN 90 | 6.77E-03 | 3.83 |
| ***IMET 1333*** | TUBULIN BETA CHAIN | 6.78E-03 | 3.12 |
| ***IMET 6620*** | T-COMPLEX PROTEIN 1 SUBUNIT BETA | 6.79E-03 | -1.22 |
| ***IMET 4621*** | NADH:UBIQUINONE OXIDOREDUCTASE COMPLEX I INTERMEDIATE-ASSOCIATED PROTEIN 30 | 6.82E-03 | -2.68 |
| ***IMET 7169*** | UNCHARACTERIZED PROTEIN | 6.83E-03 | -1.82 |
| ***IMET 9755*** | PHOTOSYSTEM I P700 CHLOROPHYLL A APOPROTEIN A1 | 6.92E-03 | -1.30 |
| ***IMET 1727*** | UNCHARACTERIZED PROTEIN | 6.99E-03 | 1.31 |
| ***IMET 8209*** | RNA BINDING PROTEIN | 7.18E-03 | 2.80 |
| ***IMET 4944*** | ATP SYNTHASE GAMMA CHAIN, CHLOROPLASTIC (ODONTELLA SINENSIS) | 7.29E-03 | -0.99 |
| ***IMET 4410*** | THREONINE SYNTHASE | 7.48E-03 | 2.00 |
| ***IMET 9488*** | V-TYPE PROTON ATPASE SUBUNIT F | 7.53E-03 | 1.90 |
| ***IMET 4519*** | TRANSKETOLASE | 7.54E-03 | -1.69 |
| ***IMET 4727*** | CHLOROPLAST SEDOHEPTULOSE --BISPHOSPHATASE | 7.55E-03 | -1.52 |
| ***IMET 8783*** | SMALL NUCLEAR RIBONUCLEOPROTEIN COMPONENT | 7.66E-03 | -1.87 |
| ***IMET 5130*** | UNCHARACTERIZED PROTEIN | 7.71E-03 | -2.09 |
| ***IMET 9834*** | ATP SYNTHASE SUBUNIT ALPHA,CHLOROPLASTIC | 8.11E-03 | -0.66 |
| ***IMET 8185*** | UNCHARACTERIZED PROTEIN | 8.14E-03 | 0.94 |
| ***IMET 5612*** | UNCHARACTERIZED PROTEIN | 8.21E-03 | -1.80 |
| ***IMET 8591*** | NUCLEAR ACID BINDING PROTEIN | 8.34E-03 | -1.71 |
| ***IMET 6191*** | 3-OXOACID TRANSFERASE 1 | 8.38E-03 | 0.88 |
| ***IMET 5886*** | PROTEIN DISULFIDE-ISOMERASE | 8.49E-03 | 1.74 |
| ***IMET 228*** | VCP1 | 8.68E-03 | -1.63 |
| ***IMET 8646*** | DTDP-GLUCOSE 4,6-DEHYDRATASE | 8.72E-03 | 1.60 |
| ***IMET 2162*** | CHALCONE ISOMERASE-LIKE PROTEIN | 8.81E-03 | 2.33 |
| ***IMET 5876*** | SIGMA 54 MODULATION PROTEIN RIBOSOMAL PROTEIN S30EA (NANNOCHLOROPSIS GADITANA) | 8.96E-03 | -1.35 |
| ***IMET 7588*** | UNCHARACTERIZED PROTEIN | 9.07E-03 | -1.45 |
| ***IMET 6185*** | KELCH REPEAT PROTEIN | 9.17E-03 | 2.46 |
| ***IMET 418*** | TRNA-SPECIFIC ADENOSINE DEAMINASE (SPIROCHAETES BACTERIUM GWB1) | 9.32E-03 | -0.82 |
| ***IMET 110*** | UNCHARACTERIZED PROTEIN | 9.46E-03 | -2.17 |
| ***IMET 100*** | GOLGI REASSEMBLY-STACKING PROTEIN 2 | 9.89E-03 | 2.09 |
| ***IMET 9365*** | UNCHARACTERIZED PROTEIN | 9.98E-03 | -2.09 |
| ***IMET 7714*** | SHORT-CHAIN DEHYDROGENASE | 1.00E-02 | -1.46 |
| ***IMET 6973*** | UNCHARACTERIZED PROTEIN | 1.02E-02 | -0.84 |
| ***IMET 4663*** | SIGNAL PEPTIDASE I | 1.02E-02 | 0.90 |
| ***IMET 4835*** | UNCHARACTERIZED PROTEIN(ECTOCARPUS SILICULOSUS) | 1.06E-02 | -1.75 |
| ***IMET 3730*** | COA-DISULFIDE REDUCTASE (SPIROCHAETES BACTERIUM RBG) | 1.06E-02 | 1.77 |
| ***IMET 9767*** | ATP-DEPENDENT ZINC METALLOPROTEASE FTSH | 1.07E-02 | -1.25 |
| ***IMET 9761*** | 50S RIBOSOMAL PROTEIN L11, CHLOROPLASTIC | 1.08E-02 | 0.96 |
| ***IMET 4757*** | ALCOHOL DEHYDROGENASE | 1.09E-02 | 1.52 |
| ***IMET 9812*** | PHOTOSYSTEM I REACTION CENTER SUBUNIT III | 1.12E-02 | -2.86 |
| ***IMET 3512*** | HYPOTHETICAL PROTEIN | 1.13E-02 | -1.60 |
| ***IMET 8312*** | PUTATIVE UNCHARACTERIZED PROTEIN ALNC14C43G3593 | 1.15E-02 | 2.09 |
| ***IMET 7611*** | BETA-ASPARTYL ASPARAGINYL FAMILY | 1.16E-02 | -2.42 |
| ***IMET 1469*** | RIBOSOMAL PROTEIN L15 | 1.17E-02 | -2.21 |
| ***IMET 9885*** | NADH-DEHYDROGENASE SUBUNIT 7 | 1.21E-02 | -1.51 |
| ***IMET 9538*** | DELTA-1-PYRROLINE-5-CARBOXYLATE SYNTHASE | 1.21E-02 | -0.69 |
| ***IMET 7882*** | PYRUVATE KINASE (NANNOCHLOROPSIS GADITANA) | 1.21E-02 | -0.70 |
| ***IMET 2271*** | PEPTIDYL-PROLYL CIS-TRANS ISOMERASE D-LIKE PROTEIN | 1.23E-02 | -1.22 |
| ***IMET 5283*** | TUBULIN ALPHA CHAIN | 1.24E-02 | 2.82 |
| ***IMET 1760*** | PHOTOSYSTEM II OXYGEN EVOLVING COMPLEX | 1.25E-02 | 1.98 |
| ***IMET 8482*** | UNCHARACTERIZED PROTEIN | 1.31E-02 | 1.63 |
| ***IMET 71*** | TRIOSEPHOSPHATEISOMERASE | 1.34E-02 | 0.65 |
| ***IMET 5785*** | 60S RIBOSOMAL PROTEIN I13 | 1.41E-02 | -1.08 |
| ***IMET 6455*** | ZEAXANTHIN EPOXIDASE 1 | 1.42E-02 | -1.73 |
| ***IMET 9866*** | HYPOTHETICAL PROTEIN | 1.49E-02 | -5.70 |
| ***IMET 6371*** | DELTA-AMINOLEVULINIC ACID DEHYDRATASE | 1.51E-02 | -1.16 |
| ***IMET 2465*** | UNCHARACTERIZED PROTEIN | 1.53E-02 | -1.14 |
| ***IMET 9179*** | 60S RIBOSOMAL PROTEIN L11 | 1.53E-02 | -0.76 |
| ***IMET 1652*** | H/ACA RIBONUCLEOPROTEIN COMPLEX SUBUNIT 4 | 1.54E-02 | -1.32 |
| ***IMET 7984*** | STRESS-INDUCED PROTEIN STI1 | 1.55E-02 | -0.23 |
| ***IMET 6520*** | ABC SUBFAMILY ABCG | 1.57E-02 | 1.66 |
| ***IMET 704*** | PHOSPHORIBOSYLFORMYLGLYCINAMIDINE SYNTHASE | 1.59E-02 | 2.31 |
| ***IMET 669*** | NADH UBIQUINONE | 1.59E-02 | -1.27 |
| ***IMET 4228*** | UNCHARACTERIZED PROTEIN | 1.59E-02 | 2.13 |
| ***IMET 2927*** | UNCHARACTERIZED PROTEIN | 1.62E-02 | -1.23 |
| ***IMET 7578*** | UNCHARACTERIZED PROTEIN | 1.62E-02 | -0.60 |
| ***IMET 7776*** | COBALAMIN SYNTHESIS PROTEIN P47K | 1.65E-02 | 1.28 |
| ***IMET 5559*** | GLUCOSAMINE--FRUCTOSE-6-PHOSPHATE AMINOTRANSFERASE | 1.66E-02 | 1.64 |
| ***IMET 2824*** | RNA-BINDING MUSASHI 2-LIKE PROTEIN | 1.67E-02 | -0.73 |
| ***IMET 8616*** | PREPROTEIN TRANSLOCASE SUBUNIT YIDC | 1.70E-02 | -1.62 |

**Table S3: Comparison of protein quantification results of +N and -N samples.** Proteins showing significant regulation between the combined +N dataset and the combined -N dataset based on a two-sample-t-test. Proteins were identified as being significantly regulated between +N and -N with a FDR-value below 0.05. The regulation factor (RF) was calculated by subtracting log2 median normalized quantification values of +N from –N.

| **Gene ID** | **Description** | **p-value between subpopulations** | **RF ((+N)-(-N))** |
| --- | --- | --- | --- |
| ***IMET 4952*** | HEATSHOCK PROTEIN 70 | 3.19E-08 | 1.96 |
| ***IMET 8093*** | PHOSPHOGLYCERATEKINASE | 5.15E-08 | 2.19 |
| ***IMET 568*** | 14-3-3-LIKEPROTEIN | 6.12E-08 | 1.96 |
| ***IMET 2218*** | HYPOTHETICALPROTEIN GLR2150 | 6.74E-08 | 2.87 |
| ***IMET 4118*** | F-TYPEH+-TRANSPORTING ATPASE OLIGOMYCIN SENSITIVITY CONFERRAL PROTEIN | 7.96E-08 | 1.93 |
| ***IMET 3544*** | FRUCTOSE-BISPHOSPHATASE | 1.39E-07 | 2.41 |
| ***IMET 3592*** | BRANCHED-CHAINAMINO ACID AMINOTRANSFERASE | 1.60E-07 | 1.88 |
| ***IMET 2623*** | EXTRINSICPROTEIN IN PHOTOSYSTEM II | 1.75E-07 | 2.51 |
| ***IMET 7547*** | CONSERVEDUNKNOWN PROTEIN | 2.10E-07 | 1.20 |
| ***IMET 8722*** | F-TYPEH+-TRANSPORTING ATPASE SUBUNIT GAMMA | 2.13E-07 | 2.02 |
| ***IMET 5914*** | HYPOTHETICALPROTEIN SORBIDRAFT_09G025900 | 2.30E-07 | 2.20 |
| ***IMET 7984*** | HYPOTHETICALPROTEIN THAOC_16820 | 5.21E-07 | 1.98 |
| ***IMET 4963*** | ATP-DEPENDENTRNA HELICASE EIF4A, PUTATIVE | 5.39E-07 | 2.45 |
| ***IMET 627*** | EF2,TRANSLATION ELONGATION FACTOR 2 | 5.48E-07 | 1.33 |
| ***IMET 4695*** | PREDICTEDPROTEIN | 5.64E-07 | 1.81 |
| ***IMET 9831*** | CLPPROTEASE ATP BINDING SUBUNIT | 6.02E-07 | 3.19 |
| ***IMET 6515*** | PREDICTEDPROTEIN | 6.68E-07 | 2.30 |
| ***IMET 9802*** | MOLECULARCHAPERONE | 6.76E-07 | 1.12 |
| ***IMET 7180*** | PHOSPHOGLYCERATEMUTASE | 8.33E-07 | 3.96 |
| ***IMET 3079*** | CONSERVEDUNKNOWN PROTEIN | 1.18E-06 | 4.18 |
| ***IMET 1582*** | PEPTIDYL-PROLYLCIS-TRANS ISOMERASE | 1.19E-06 | 2.70 |
| ***IMET 7071*** | BETAINEALDEHYDE DEHYDROGENASE 2 | 1.33E-06 | 3.37 |
| ***IMET 3797*** | 60SRIBOSOMAL PROTEIN L7A-2 | 1.56E-06 | 3.55 |
| ***IMET 6283*** | CONSERVEDUNKNOWN PROTEIN | 1.66E-06 | 1.98 |
| ***IMET 9880*** | CYTOCHROMEC553 | 1.68E-06 | 2.26 |
| ***IMET 6985*** | HYPOTHETICALPROTEIN | 1.99E-06 | 3.20 |
| ***IMET 8104*** | LL-DIAMINOPIMELATEAMINOTRANSFERASE | 2.27E-06 | 1.81 |
| ***IMET 4800*** | F-TYPEH+-TRANSPORTING ATPASE SUBUNIT BETA, PARTIAL | 3.79E-06 | 1.22 |
| ***IMET 1548*** | PRE-MRNA-PROCESSINGFACTOR 17 | 4.31E-06 | 2.13 |
| ***IMET 1575*** | GLYCERALDEHYDE-3-PHOSPHATEDEHYDROGENASE | 4.44E-06 | 2.60 |
| ***IMET 6084*** | EUKARYOTICINITIATION FACTOR 4A-III, PUTATIVE | 4.95E-06 | 1.82 |
| ***IMET 3336*** | CONSERVEDUNKNOWN PROTEIN | 4.96E-06 | 1.59 |
| ***IMET 4983*** | HYPOTHETICALPROTEIN OSJ_01299 | 5.21E-06 | 1.68 |
| ***IMET 614*** | PREDICTEDPROTEIN | 5.59E-06 | 1.42 |
| ***IMET 5915*** | HEATSHOCK PROTEIN HSP20 | 6.10E-06 | 4.72 |
| ***IMET 5419*** | PEPTIDYLPROLYLISOMERASE A | 6.45E-06 | 2.70 |
| ***IMET 2578*** | HYPOTHETICALPROTEIN AURANDRAFT_33383 | 6.85E-06 | 1.83 |
| ***IMET 71*** | TRIOSEPHOSPHATEISOMERASE | 7.70E-06 | 1.82 |
| ***IMET 4340*** | HYPOTHETICALPROTEIN NGA_0714200 | 8.66E-06 | 3.31 |
| ***IMET 9089*** | HYPOTHETICALPROTEIN PHYSODRAFT_353904 | 8.70E-06 | 1.40 |
| ***IMET 6830*** | 40SRIBOSOMAL PROTEIN S13 | 8.91E-06 | 1.67 |
| ***IMET 2612*** | FERREDOXIN--NADP+REDUCTASE | 8.93E-06 | 1.28 |
| ***IMET 8161*** | ALDEHYDEDEHYDROGENASE FAMILY 7 MEMBER A1 | 8.98E-06 | 2.86 |
| ***IMET 3227*** | PYRUVATE,PHOSPHATE DIKINASE | 9.49E-06 | 1.53 |
| ***IMET 9452*** | PREDICTEDPROTEIN | 9.75E-06 | 1.65 |
| ***IMET 8110*** | SHORTCHAIN DEHYDROGENASE | 1.02E-05 | 1.20 |
| ***IMET 3410*** | HYPOTHETICALPROTEIN GUITHDRAFT_83873, PARTIAL | 1.06E-05 | 3.25 |
| ***IMET 9903*** | ATPSYNTHASE F1 SUBUNIT ALPHA | 1.09E-05 | 1.38 |
| ***IMET 9775*** | ELONGATIONFACTOR TU | 1.23E-05 | 1.78 |
| ***IMET 1813*** | HYPOTHETICALPROTEIN AURANDRAFT_52498 | 1.40E-05 | 1.12 |
| ***IMET 4411*** | HYPOTHETICALPROTEIN PHYSODRAFT_355326 | 1.47E-05 | 2.08 |
| ***IMET 1227*** | HYPOTHETICALPROTEIN FOMMEDRAFT_16036 | 1.54E-05 | 3.78 |
| ***IMET 8619*** | HYPOTHETICALPROTEIN NGA_0605600 | 1.79E-05 | 2.59 |
| ***IMET 3390*** | ASPARTYLTRNASYNTHETASE PUTATIVE | 1.82E-05 | 1.17 |
| ***IMET 2557*** | VACUOLARATPASE | 1.87E-05 | 0.80 |
| ***IMET 4190*** | HYPOTHETICALPROTEIN NGA_0386800 | 1.92E-05 | 1.95 |
| ***IMET 8889*** | THIOREDOXINF | 2.05E-05 | 2.24 |
| ***IMET 7234*** | HYPOTHETICALPROTEIN AURANDRAFT_37826 | 2.06E-05 | 1.11 |
| ***IMET 1261*** | HYPOTHETICALPROTEIN PHYSODRAFT_353332 | 2.27E-05 | 2.47 |
| ***IMET 2452*** | CHOLINEDEHYDROGENASE | 2.36E-05 | 1.75 |
| ***IMET 2802*** | HYPOTHETICALPROTEIN GUITHDRAFT_68651 | 2.51E-05 | 1.83 |
| ***IMET 4728*** | NOANNOTATION | 2.58E-05 | 3.30 |
| ***IMET 4312*** | CONSERVEDUNKNOWN PROTEIN | 2.58E-05 | 2.98 |
| ***IMET 8976*** | ARGININOSUCCINATESYNTHETASE | 2.60E-05 | 2.10 |
| ***IMET 1508*** | NOANNOTATION | 2.95E-05 | 2.29 |
| ***IMET 7335*** | ACETYLORNITHINEDEACETYLASE | 3.16E-05 | 3.04 |
| ***IMET 5865*** | GLYCINEHYDROXYMETHYLTRANSFERASE | 3.20E-05 | 2.13 |
| ***IMET 5794*** | PHOSPHOGLYCERATEKINASE | 3.26E-05 | 2.06 |
| ***IMET 1963*** | FERREDOXIN-THIOREDOXINREDUCTASE, CATALYTIC CHAIN | 3.50E-05 | 1.28 |
| ***IMET 4792*** | TRANS-2-ENOYL-COAREDUCTASE | 3.61E-05 | 2.56 |
| ***IMET 8915*** | GTP-BINDINGPROTEIN SAR1 | 3.75E-05 | 1.25 |
| ***IMET 1606*** | GLUTATHIONEREDUCTASE | 4.26E-05 | 3.83 |
| ***IMET 1760*** | PREDICTEDPROTEIN | 4.47E-05 | 3.41 |
| ***IMET 2084*** | PHOTOSYSTEMII OXYGEN-EVOLVING ENHANCER PROTEIN 1 | 4.84E-05 | 1.77 |
| ***IMET 4319*** | CONSERVEDUNKNOWN PROTEIN | 4.99E-05 | 2.57 |
| ***IMET 6190*** | PEROXIREDOXINQ | 5.22E-05 | 2.85 |
| ***IMET 5871*** | HYPOTHETICALPROTEIN AURANDRAFT_70645 | 5.35E-05 | 1.02 |
| ***IMET 59*** | 5-OXOPROLINASE | 5.45E-05 | 3.35 |
| ***IMET 9519*** | MOLECULARCHAPERONE HTPG | 5.47E-05 | 0.86 |
| ***IMET 74*** | HYPOTHETICALPROTEIN NGA_0015702 | 5.61E-05 | 3.67 |
| ***IMET 9792*** | 30SRIBOSOMAL PROTEIN S17 | 5.72E-05 | 1.96 |
| ***IMET 1994*** | DJ-1FAMILY PROTEIN | 5.73E-05 | 3.07 |
| ***IMET 2465*** | NOANNOTATION | 6.10E-05 | 2.27 |
| ***IMET 5208*** | N-ACETYL-GAMMA-GLUTAMYL-PHOSPHATE/N-ACETYL-GAMMA-AMINOADIPYL-PHOSPHATEREDUCTASE | 6.58E-05 | 1.49 |
| ***IMET 4634*** | RIBULOSE-PHOSPHATE3-EPIMERASE | 6.67E-05 | 1.96 |
| ***IMET 126*** | CONSERVEDHYPOTHETICAL PROTEIN | 6.74E-05 | -1.29 |
| ***IMET 9879*** | PHOTOSYSTEMII CYTOCHROME C550 | 7.00E-05 | 1.74 |
| ***IMET 3847*** | HYPOTHETICALPROTEIN THAOC_04380 | 7.27E-05 | 2.01 |
| ***IMET 611*** | HEATSHOCK PROTEIN 70 PUTATIVE | 7.39E-05 | 1.68 |
| ***IMET 4270*** | OXIDOREDUCTASEDOMAIN PROTEIN, PARTIAL | 7.42E-05 | 1.17 |
| ***IMET 9549*** | CRAL/TRIODOMAIN CONTAINING PROTEIN | 7.69E-05 | 1.08 |
| ***IMET 2436*** | GLUTAMATEDEHYDROGENASE 1 | 7.77E-05 | 1.19 |
| ***IMET 3619*** | HYPOTHETICALPROTEIN NGA_0361402 | 8.21E-05 | 2.92 |
| ***IMET 2449*** | HYPOTHETICALPROTEIN NGA_0429000 | 8.31E-05 | -1.55 |
| ***IMET 4156*** | ARGONAUTE1, PARTIAL | 8.61E-05 | 2.41 |
| ***IMET 7714*** | HYPOTHETICALPROTEIN GUITHDRAFT_152655 | 8.70E-05 | 2.34 |
| ***IMET 3630*** | HYPOTHETICALPROTEIN NGA_0524200 | 9.32E-05 | 1.28 |
| ***IMET 3595*** | SMALLSUBUNIT RIBOSOMAL PROTEIN S7E | 9.32E-05 | 2.54 |
| ***IMET 9834*** | ATPSYNTHASE CF1 ALPHA SUBUNIT | 9.49E-05 | 1.24 |
| ***IMET 4821*** | SMALLSUBUNIT RIBOSOMAL PROTEIN S8E, PARTIAL | 1.09E-04 | 2.29 |
| ***IMET 3450*** | NICOTINANAMINEAMINOTRANSFERASE A | 1.11E-04 | 1.50 |
| ***IMET 8222*** | NOANNOTATION | 1.13E-04 | 3.54 |
| ***IMET 4633*** | PUTATIVEPLASTID LIGHT HARVESTING PROTEIN ISOFORM 10 | 1.15E-04 | 2.97 |
| ***IMET 2560*** | CONSERVEDUNKNOWN PROTEIN | 1.17E-04 | 3.24 |
| ***IMET 142*** | CONSERVEDUNKNOWN PROTEIN | 1.19E-04 | 1.63 |
| ***IMET 1274*** | ENOLASE | 1.20E-04 | 1.84 |
| ***IMET 2173*** | NOANNOTATION | 1.22E-04 | 2.96 |
| ***IMET 9241*** | GLYOXALASEDOMAIN-CONTAINING 4-LIKE PROTEIN | 1.25E-04 | 3.69 |
| ***IMET 4410*** | THREONINESYNTHASE | 1.27E-04 | 3.12 |
| ***IMET 2617*** | EF-1GUANINE NUCLEOTIDE EXCHANGE DOMAIN-CONTAINING | 1.32E-04 | 1.72 |
| ***IMET 4835*** | NOANNOTATION | 1.41E-04 | 2.64 |
| ***IMET 2534*** | PREDICTEDPROTEIN | 1.45E-04 | 3.21 |
| ***IMET_9885*** | CYTOCHROMEC OXIDASE SUBUNIT II | 1.48E-04 | 2.31 |
| ***IMET 2480*** | FERREDOXINCOMPONENT | 1.51E-04 | 0.99 |
| ***IMET 5459*** | CONSERVEDUNKNOWN PROTEIN | 1.60E-04 | 2.88 |
| ***IMET 7884*** | CONSERVEDUNKNOWN PROTEIN | 1.67E-04 | 1.38 |
| ***IMET 4807*** | VACUOLARSORTING PROTEIN 35, PARTIAL | 1.69E-04 | 1.99 |
| ***IMET 9359*** | CONSERVEDUNKNOWN PROTEIN | 1.70E-04 | 4.93 |
| ***IMET 4755*** | ACYLDEHYDRATASE | 1.84E-04 | 3.07 |
| ***IMET 4874*** | POTASSIUM/SODIUMEFFLUX P-TYPE ATPASE | 1.89E-04 | 1.41 |
| ***IMET 9194*** | 40SRIBOSOMAL PROTEIN S4 | 1.97E-04 | 1.47 |
| ***IMET 9859*** | PHOTOSYSTEMI REACTION CENTER SUBUNIT II | 1.97E-04 | 1.37 |
| ***IMET 8077*** | HYPOTHETICALPROTEIN PHYSODRAFT_354972 | 2.03E-04 | 1.06 |
| ***IMET 1210*** | CONSERVEDCARBOHYDRATE BINDING PROTEIN | 2.09E-04 | 1.76 |
| ***IMET 5304*** | LIGHTHARVESTING COMPLEX PROTEIN | 2.10E-04 | 1.77 |
| ***IMET 7595*** | HYPOTHETICALPROTEIN THAOC_09685 | 2.13E-04 | 2.79 |
| ***IMET 3362*** | EUKARYOTICELONGATION FACTOR-1 B GAMMA | 2.19E-04 | 0.93 |
| ***IMET 7925*** | DNA-BINDINGPROTEIN HU | 2.25E-04 | 2.65 |
| ***IMET 5055*** | PEROXIREDOXIN5, ATYPICAL 2-CYS PEROXIREDOXIN | 2.28E-04 | 1.90 |
| ***IMET 3651*** | HYPOTHETICALPROTEIN PHYSODRAFT_330563 | 2.36E-04 | 2.66 |
| ***IMET 4771*** | EXTRINSICPROTEIN IN PHOTOSYSTEM II | 2.43E-04 | 1.78 |
| ***IMET 7833*** | PHOSPHOGLYCERATEKINASE | 2.47E-04 | 1.95 |
| ***IMET 8782*** | CONSERVEDUNKNOWN PROTEIN | 2.49E-04 | 1.94 |
| ***IMET 2114*** | CONSERVEDUNKNOWN PROTEIN | 2.53E-04 | 2.74 |
| ***IMET 7688*** | CHAPERONEPROTEIN | 2.62E-04 | 1.62 |
| ***IMET 2835*** | UDP-GLUCOSE4-EPIMERASE | 2.68E-04 | 1.43 |
| ***IMET 1674*** | HYPOTHETICALPROTEIN THAOC_24894 | 2.73E-04 | 1.19 |
| ***IMET 4727*** | SEDOHEPTULOSE-BISPHOSPHATASE | 3.02E-04 | 2.00 |
| ***IMET 4984*** | HYPOTHETICALPROTEIN GUITHDRAFT_158188 | 3.05E-04 | 1.75 |
| ***IMET 5441*** | HYPOTHETICALPROTEIN NGA_0174300 | 3.15E-04 | 1.25 |
| ***IMET 2483*** | ATPASE | 3.24E-04 | 1.60 |
| ***IMET 2498*** | GLUTATHIONEPEROXIDASE PUTATIVE | 3.24E-04 | 0.93 |
| ***IMET 4418*** | PUTATIVEBIFUNCTIONAL P-450/NADPH-P450 REDUCTASE 1 | 3.27E-04 | 0.95 |
| ***IMET 4293*** | HYPOTHETICALPROTEIN NGA_0175600 | 3.32E-04 | 1.97 |
| ***IMET 646*** | CONSERVEDUNKNOWN PROTEIN | 3.38E-04 | 2.41 |
| ***IMET 4400*** | HISTONE | 3.41E-04 | 2.37 |
| ***IMET 9869*** | CYTOCHROMEB559 BETA CHAIN | 3.50E-04 | -1.68 |
| ***IMET 5281*** | PHOSPHOADENYLYL-SULFATEREDUCTASE | 3.53E-04 | 1.71 |
| ***IMET 706*** | HYPOTHETICALPROTEIN PHYSODRAFT_293395 | 3.62E-04 | 0.84 |
| ***IMET 1200*** | CONSERVEDUNKNOWN PROTEIN | 3.72E-04 | 3.24 |
| ***IMET 6428*** | MALONYL-COA:ACPTRANSACYLASE | 3.83E-04 | 2.21 |
| ***IMET 2281*** | LARGESUBUNIT RIBOSOMAL PROTEIN L6E | 3.92E-04 | 1.24 |
| ***IMET 8460*** | LARGESUBUNIT RIBOSOMAL PROTEIN L27E | 3.99E-04 | 3.00 |
| ***IMET 2792*** | CONSERVEDUNKNOWN PROTEIN | 4.02E-04 | 1.43 |
| ***IMET 4175*** | PREDICTEDPROTEIN | 4.11E-04 | 1.99 |
| ***IMET 4139*** | LARGESUBUNIT RIBOSOMAL PROTEIN L22E | 4.27E-04 | 1.67 |
| ***IMET 9836*** | NOANNOTATION | 4.28E-04 | 2.82 |
| ***IMET 1999*** | LARGESUBUNIT RIBOSOMAL PROTEIN L5E | 4.37E-04 | 3.28 |
| ***IMET 6337*** | SIMILARTO DEK ONCOGENE | 4.56E-04 | 1.85 |
| ***IMET 1620*** | NADH:UBIQUINONEOXIDOREDUCTASE COMPLEX I INTERMEDIATE-ASSOCIATED PROTEIN 30 | 4.62E-04 | 0.78 |
| ***IMET 5785*** | LARGESUBUNIT RIBOSOMAL PROTEIN L13E | 4.64E-04 | 1.43 |
| ***IMET 3690*** | TRANSLATIONALLYCONTROLLED TUMOR PROTEIN | 4.65E-04 | 3.80 |
| ***IMET 7651*** | CONSERVEDUNKNOWN PROTEIN | 4.66E-04 | -1.04 |
| ***IMET 5030*** | PYRUVATEDEHYDROGENASE | 4.82E-04 | -2.69 |
| ***IMET 9538*** | HYPOTHETICALPROTEIN PHYSODRAFT_555969 | 4.85E-04 | -0.88 |
| ***IMET 985*** | 2-CYSPEROXIREDOXIN | 5.02E-04 | 3.04 |
| ***IMET 9356*** | ASPARTATE-SEMIALDEHYDEDEHYDROGENASE | 5.20E-04 | 1.07 |
| ***IMET 3260*** | 3-HYDROXYISOBUTYRATEDEHYDROGENASE | 5.34E-04 | 1.98 |
| ***IMET 8076*** | SMALLSUBUNIT RIBOSOMAL PROTEIN S3E | 5.73E-04 | 2.05 |
| ***IMET 157*** | DIHYDROLIPOAMIDEDEHYDROGENASE | 5.75E-04 | 1.45 |
| ***IMET 9767*** | ATP-DEPENDENTMETALLOPROTEASE | 5.96E-04 | 1.58 |
| ***IMET 9873*** | PHOTOSYSTEMI SUBUNIT XI | 5.99E-04 | 3.60 |
| ***IMET 2931*** | PHOSPHORIBULOKINASE | 6.24E-04 | 1.19 |
| ***IMET 6219*** | HYPOTHETICALPROTEIN AURANDRAFT_72012 | 6.34E-04 | 1.53 |
| ***IMET 5949*** | TRANSLATIONELONGATION FACTOR TS | 6.40E-04 | 1.49 |
| ***IMET 5310*** | BETA-D-GALACTOSIDASE | 6.56E-04 | 1.40 |
| ***IMET 5190*** | HYPOTHETICALPROTEIN PHYSODRAFT_545625 | 6.58E-04 | 1.12 |
| ***IMET 478*** | LIGHTHARVESTING COMPLEX PROTEIN | 6.70E-04 | 1.34 |
| ***IMET 3762*** | PEROXIREDOXIN1, PARTIAL | 6.78E-04 | 1.99 |
| ***IMET 5482*** | UNNAMEDPROTEIN PRODUCT | 6.83E-04 | 1.40 |
| ***IMET 9373*** | CONSERVEDUNKNOWN PROTEIN | 6.99E-04 | 0.69 |
| ***IMET 9807*** | MAGNESIUM-PROTOPORPHYRINIX MONOMETHYL ESTER AEROBIC OXIDATIVE CYCLASE | 7.01E-04 | 2.33 |
| ***IMET 404*** | PYRROLINE-5-CARBOXYLATEREDUCTASE | 7.06E-04 | 0.94 |
| ***IMET 8757*** | PLASTIDDIVISION PROTEIN FTSZ | 7.18E-04 | 1.20 |
| ***IMET 1322*** | HYPOTHETICALPROTEIN PHYSODRAFT_557596 | 7.25E-04 | 1.44 |
| ***IMET 7516*** | CONSERVEDUNKNOWN PROTEIN | 7.26E-04 | 1.20 |
| ***IMET 4839*** | PHOSPHOENOLPYRUVATECARBOXYLASE | 7.50E-04 | 1.18 |
| ***IMET 4694*** | HYPOTHETICALPROTEIN AURANDRAFT_72012 | 7.52E-04 | 1.44 |
| ***IMET 1214*** | PEROXISOMALMEMBRANE MPV17/PMP22-LIKE PROTEIN | 7.60E-04 | 3.82 |
| ***IMET 650*** | CONSERVEDUNKNOWN PROTEIN | 7.65E-04 | 1.89 |
| ***IMET 2899*** | MEMBRANEALANYL AMINOPEPTIDASE | 7.73E-04 | 1.57 |
| ***IMET 4437*** | HYPOTHETICALPROTEIN GUITHDRAFT_98620 | 8.08E-04 | 2.23 |
| ***IMET 1605*** | BETA-KETOACYL-COATHIOLASE | 8.17E-04 | 2.22 |
| ***IMET 2621*** | NUCLEOSIDE-DIPHOSPHATEKINASE | 8.28E-04 | 0.59 |
| ***IMET 5113*** | 20SPROTEASOME SUBUNIT ALPHA 1 | 8.50E-04 | 1.22 |
| ***IMET 7588*** | CONSERVEDUNKNOWN PROTEIN | 8.50E-04 | 1.67 |
| ***IMET 5417*** | CHLOROPLAST3-OXOACYL- | 8.61E-04 | 1.95 |
| ***IMET 1484*** | NOANNOTATION | 8.66E-04 | 2.26 |
| ***IMET 626*** | MITOCHONDRIALPROCESSING PEPTIDASE ALPHA SUBUNIT | 9.09E-04 | 1.41 |
| ***IMET 1687*** | CONSERVEDUNKNOWN PROTEIN | 9.11E-04 | 2.26 |
| ***IMET 1882*** | HYPOTHETICALPROTEIN THAOC_34340 | 9.41E-04 | 1.45 |
| ***IMET 3052*** | HYPOTHETICALPROTEIN CHLNCDRAFT_59634 | 9.68E-04 | 1.59 |
| ***IMET 3512*** | HYPOTHETICALPROTEIN THAOC_26649 | 9.88E-04 | 1.99 |
| ***IMET 9790*** | 50SRIBOSOMAL PROTEIN L5 | 9.98E-04 | -0.74 |
| ***IMET 1749*** | HYPOTHETICALPROTEIN NGA_0477710 | 1.01E-03 | 0.97 |
| ***IMET 8724*** | PYRUVATEDEHYDROGENASE E2 COMPONENT | 1.03E-03 | 1.31 |
| ***IMET 9835*** | NOANNOTATION | 1.05E-03 | 1.22 |
| ***IMET 3263*** | OXIDOREDUCTASE,NAD-BINDING, MYO-INOSITOL 2-DEHYDROGENASE | 1.07E-03 | 1.44 |
| ***IMET 2508*** | ACYL-COADEHYDROGENASE | 1.10E-03 | -1.27 |
| ***IMET 2845*** | SUPEROXIDEDISMUTASE | 1.11E-03 | 2.11 |
| ***IMET 4918*** | FLAGELLARASSOCIATED PROTEIN | 1.14E-03 | 0.92 |
| ***IMET 9696*** | TRIOSEPHOSPHATEISOMERASE/GLYCERALDEHYDE3PHOSPHATE DEHYDROGENASE PUTATIVE | 1.17E-03 | 1.49 |
| ***IMET 3351*** | SMALLSUBUNIT RIBOSOMAL PROTEIN S12E | 1.21E-03 | 3.25 |
| ***IMET 1886*** | PREDICTED:GLUTATHIONE PEROXIDASE-LIKE | 1.24E-03 | 2.47 |
| ***IMET 4803*** | HYPOTHETICALPROTEIN NGA_2003000, PARTIAL | 1.25E-03 | 2.27 |
| ***IMET 8653*** | HYPOTHETICALPROTEIN GUITHDRAFT_64569 | 1.26E-03 | 2.44 |
| ***IMET 1911*** | ATP-CITRATESYNTHASE | 1.27E-03 | 1.27 |
| ***IMET 8972*** | 6,7-DIMETHYL-8-RIBITYLLUMAZINESYNTHASE | 1.28E-03 | 2.67 |
| ***IMET 7063*** | HYPOTHETICALPROTEIN PHYSODRAFT_354972 | 1.32E-03 | 1.66 |
| ***IMET 5612*** | LIGHTHARVESTING COMPLEX PROTEIN 2 | 1.34E-03 | 2.38 |
| ***IMET 5508*** | NOANNOTATION | 1.35E-03 | 2.26 |
| ***IMET 5528*** | TRIGGERFACTOR | 1.35E-03 | 1.78 |
| ***IMET 8438*** | T-COMPLEXPROTEIN 1 SUBUNIT THETA | 1.35E-03 | 0.92 |
| ***IMET 8842*** | SIMILARTO 3-PHOSPHOADENOSINE 5-PHOSPHOSULFATE SYNTHASE 2 ISOFORM 2 | 1.36E-03 | -0.78 |
| ***IMET 1764*** | CONSERVEDUNKNOWN PROTEIN | 1.42E-03 | 1.29 |
| ***IMET 8847*** | NOANNOTATION | 1.44E-03 | 1.41 |
| ***IMET 6049*** | PREDICTEDPROTEIN | 1.48E-03 | 0.88 |
| ***IMET 8583*** | PYRUVATEKINASE, PARTIAL | 1.51E-03 | 1.36 |
| ***IMET 6653*** | EEF1A2,EUKARYOTIC TRANSLATION ELONGATION FACTOR 1 ALPHA | 1.54E-03 | 1.22 |
| ***IMET 7322*** | HYPOTHETICALPROTEIN PHYSODRAFT_285408 | 1.55E-03 | 2.34 |
| ***IMET 5303*** | LIGHTHARVESTING COMPLEX PROTEIN | 1.58E-03 | 2.07 |
| ***IMET 4664*** | SIGNALPEPTIDASE, ENDOPLASMIC RETICULUM-TYPE | 1.63E-03 | 0.96 |
| ***IMET 9768*** | PHOTOSYSTEMI REACTION CENTER SUBUNIT IV | 1.63E-03 | 3.35 |
| ***IMET 8733*** | 40SRIBOSOMAL PROTEIN S15A | 1.67E-03 | 1.68 |
| ***IMET 2162*** | CHALCONEISOMERASE-LIKE PROTEIN | 1.68E-03 | 2.48 |
| ***IMET 7883*** | PHEOPHORBIDEA OXYGENASE, PUTATIVE CHLOROPLAST PRECURSOR | 1.74E-03 | 1.68 |
| ***IMET 3326*** | AMINOPEPTIDASEN | 1.78E-03 | 2.02 |
| ***IMET 6888*** | CONSERVEDUNKNOWN PROTEIN | 1.82E-03 | 2.00 |
| ***IMET 2927*** | CONSERVEDUNKNOWN PROTEIN | 1.86E-03 | 1.66 |
| ***IMET 1725*** | PREDICTEDPROTEIN | 2.02E-03 | 1.97 |
| ***IMET 9488*** | CONSERVEDUNKNOWN PROTEIN | 2.03E-03 | 2.18 |
| ***IMET 4470*** | HYPOTHETICALPROTEIN LOC100194262 | 2.11E-03 | 0.73 |
| ***IMET 4000*** | INOSITOL2-DEHYDROGENASE | 2.13E-03 | 3.33 |
| ***IMET 4166*** | KETOL-ACIDREDUCTOISOMERASE | 2.15E-03 | 0.90 |
| ***IMET 3455*** | HYPOTHETICALPROTEIN AURANDRAFT_59995 | 2.16E-03 | 1.57 |
| ***IMET 2504*** | LIGHTHARVESTING COMPLEX PROTEIN | 2.17E-03 | 1.57 |
| ***IMET 5268*** | HYPOTHETICALPROTEIN PHYSODRAFT_352502 | 2.20E-03 | 1.06 |
| ***IMET 5950*** | PREDICTEDPROTEIN | 2.26E-03 | 1.13 |
| ***IMET 7986*** | CONSERVEDUNKNOWN PROTEIN | 2.27E-03 | 1.13 |
| ***IMET 107*** | PREDICTEDPROTEIN | 2.29E-03 | 1.32 |
| ***IMET 6318*** | THIOREDOXIN-RELATEDPROTEIN | 2.29E-03 | 2.76 |
| ***IMET 4396*** | PUTATIVE3-KETOACYL-COA REDUCTASE | 2.30E-03 | 2.24 |
| ***IMET 7791*** | LONG-CHAINACYL-COENZYME A SYNTHETASE | 2.35E-03 | 0.60 |
| ***IMET 3730*** | COA-DISULFIDEREDUCTASE | 2.36E-03 | -2.43 |
| ***IMET 9077*** | HYPOTHETICALPROTEIN PTSG_03958 | 2.39E-03 | 3.40 |
| ***IMET 838*** | LIGHT-DEPENDENTNADPH:PROTOCHLOROPHYLLIDE OXIDOREDUCTASE | 2.42E-03 | -1.13 |
| ***IMET 316*** | PREDICTEDPROTEIN | 2.44E-03 | -1.56 |
| ***IMET 7464*** | CONSERVEDUNKNOWN PROTEIN | 2.48E-03 | 1.50 |
| ***IMET 3408*** | UREASEACCESSORY PROTEIN UREG | 2.68E-03 | 2.33 |
| ***IMET 116*** | PHOSPHORIBOSYLGLYCINAMIDESYNTHETASE, PUTATIVE | 2.78E-03 | 2.14 |
| ***IMET 9804*** | PHOTOSYSTEMII PROTEIN D2 | 2.89E-03 | 1.22 |
| ***IMET 5856*** | CHLOROPLASTCLP PROTEASE P | 2.99E-03 | 0.80 |
| ***IMET 7618*** | T-COMPLEXPROTEIN 1, GAMMA SUBUNIT | 3.00E-03 | 1.23 |
| ***IMET 91*** | HYPOTHETICALPROTEIN PHYSODRAFT_542262 | 3.12E-03 | 1.51 |
| ***IMET 9179*** | CONSERVEDUNKNOWN PROTEIN | 3.12E-03 | 0.89 |
| ***IMET 416*** | MALATESYNTHASE | 3.28E-03 | 2.33 |
| ***IMET 1256*** | CONSERVEDHYPOTHETICAL PROTEIN | 3.38E-03 | 2.43 |
| ***IMET 9822*** | HYPOTHETICALPROTEIN NAGA_1CHLOROPLAST82 | 3.47E-03 | 1.16 |
| ***IMET 4284*** | CONSERVEDUNKNOWN PROTEIN | 3.47E-03 | 1.47 |
| ***IMET 7311*** | HYPOTHETICALPROTEIN NGA_2114600 | 3.60E-03 | 0.94 |
| ***IMET 4432*** | L-ASCORBATEPEROXIDASE | 3.68E-03 | 2.09 |
| ***IMET 9766*** | RUBISCOEXPRESSION PROTEIN | 3.69E-03 | 1.81 |
| ***IMET 1149*** | NOANNOTATION | 3.76E-03 | 2.22 |
| ***IMET 4496*** | 40SRIBOSOMAL PROTEIN S3A-2 | 3.82E-03 | 0.92 |
| ***IMET 3426*** | INOSITOL2-DEHYDROGENASE | 3.83E-03 | 1.37 |
| ***IMET 8974*** | SOULHEME-BINDING PROTEIN | 3.85E-03 | 0.70 |
| ***IMET 5130*** | HYPOTHETICALPROTEIN NGA_0514700 | 3.92E-03 | 1.91 |
| ***IMET 8040*** | HYPOTHETICALPROTEIN AURANDRAFT_59824 | 3.94E-03 | 0.66 |
| ***IMET 6011*** | ZETA-CAROTENEDESATURASE, CHLOROPLAST PRECURSOR | 3.95E-03 | 2.45 |
| ***IMET 4052*** | MOLECULARCHAPERONES GRP78/BIP/KAR2, HSP70 SUPERFAMILY | 3.99E-03 | 0.56 |
| ***IMET 473*** | CONSERVEDUNKNOWN PROTEIN | 4.03E-03 | 2.11 |
| ***IMET 1462*** | PREDICTEDPROTEIN | 4.09E-03 | 2.51 |
| ***IMET 4621*** | NADH:UBIQUINONEOXIDOREDUCTASE COMPLEX I INTERMEDIATE-ASSOCIATED PROTEIN 30 | 4.09E-03 | 2.49 |
| ***IMET 2567*** | 3-HYDROXYACYL-COADEHYDROGENASE | 4.10E-03 | 1.27 |
| ***IMET 9761*** | 50SRIBOSOMAL PROTEIN L11 | 4.24E-03 | 0.96 |
| ***IMET 3044*** | CONSERVEDUNKNOWN PROTEIN | 4.37E-03 | 1.49 |
| ***IMET 7543*** | PUTATIVESEC61/SECY | 4.49E-03 | 0.86 |
| ***IMET 5201*** | AP-1COMPLEX SUBUNIT BETA-1 | 4.53E-03 | 0.72 |
| ***IMET 4654*** | AMINOACID ADENYLATION DOMAIN PROTEIN | 4.61E-03 | 0.96 |
| ***IMET 6038*** | HYPOTHETICALPROTEIN NGA_0437801 | 4.77E-03 | 2.31 |
| ***IMET 9772*** | PHOTOSYSTEMII 47 KDA PROTEIN | 4.86E-03 | 1.05 |
| ***IMET 8816*** | PREDICTEDPROTEIN | 4.88E-03 | 2.11 |
| ***IMET 9848*** | ATPSYNTHASE CF1 BETA SUBUNIT | 4.93E-03 | 1.08 |
| ***IMET 9840*** | 30SRIBOSOMAL PROTEIN S2 | 5.18E-03 | -0.98 |
| ***IMET 4204*** | FOX2PROTEIN | 5.22E-03 | 1.78 |
| ***IMET 7302*** | NOANNOTATION | 5.31E-03 | 1.76 |
| ***IMET 883*** | HYPOTHETICALPROTEIN PHYSODRAFT_353780 | 5.33E-03 | 1.27 |
| ***IMET 3054*** | EUKARYOTICINITIATION FACTOR 2 BETA SUBUNIT | 5.36E-03 | 0.85 |
| ***IMET 2054*** | HYPOTHETICALPROTEIN AURANDRAFT_54227 | 5.37E-03 | 1.27 |
| ***IMET 7069*** | RIBOSOMALPROTEIN L18 | 5.44E-03 | 0.99 |
| ***IMET 4454*** | CHAPERONE,DNAJ-LIKE PROTEIN | 5.57E-03 | 0.94 |
| ***IMET 9762*** | 50SRIBOSOMAL PROTEIN L1 | 5.74E-03 | 0.46 |
| ***IMET 4021*** | HYPOTHETICALPROTEIN PHYSODRAFT_361923 | 5.77E-03 | 2.01 |
| ***IMET 7889*** | HYPOTHETICALPROTEIN AURANDRAFT_69702, PARTIAL | 5.81E-03 | 1.42 |
| ***IMET 4265*** | SPLICINGFACTOR 3A SUBUNIT 3 | 5.92E-03 | 1.78 |
| ***IMET 1510*** | CONSERVEDHYPOTHETICAL PROTEIN | 6.00E-03 | 1.11 |
| ***IMET 9*** | HEATSHOCK PROTEIN HSP20 | 6.05E-03 | 3.94 |
| ***IMET 6730*** | TRIFUNCTIONALENZYME SUBUNIT ALPHA | 6.18E-03 | 1.33 |
| ***IMET 7728*** | CONSERVEDUNKNOWN PROTEIN | 6.33E-03 | 1.14 |
| ***IMET 8873*** | CYTOCHROMEB6-F COMPLEX IRON-SULFUR SUBUNIT | 6.48E-03 | 2.95 |
| ***IMET 629*** | GLUCOSE-6-PHOSPHATEISOMERASE | 6.53E-03 | 2.94 |
| ***IMET 5284*** | PREDICTEDPROTEIN | 6.59E-03 | 1.25 |
| ***IMET 310*** | HYPOTHETICALPROTEIN NGA_0432801 | 6.59E-03 | 1.63 |
| ***IMET 156*** | PEFG,PLASTID TRANSLATION ELONGATION FACTOR EF-G | 6.59E-03 | 1.47 |
| ***IMET 588*** | URATEOXIDASE | 6.66E-03 | 1.54 |
| ***IMET 4834*** | PREDICTEDPROTEIN | 6.70E-03 | 1.21 |
| ***IMET 7856*** | TRANSLATIONINITIATION FACTOR 3 SUBUNIT G | 6.71E-03 | -0.94 |
| ***IMET 673*** | CONSERVEDUNKNOWN PROTEIN | 6.79E-03 | 1.69 |
| ***IMET 9281*** | LIGHTHARVESTING COMPLEX PROTEIN | 6.81E-03 | -1.41 |
| ***IMET 1590*** | HYPOTHETICALPROTEIN PHYSODRAFT_496117 | 6.93E-03 | 0.65 |
| ***IMET 9096*** | HYPOTHETICALPROTEIN AURANDRAFT_69840 | 7.06E-03 | 1.37 |
| ***IMET 6309*** | PHOSPHORIBOSYLAMINOIMIDAZOLECARBOXAMIDEFORMYLTRANSFERASE / IMP CYCLOHYDROLASE, PARTIAL | 7.06E-03 | 1.90 |
| ***IMET 1832*** | CBBXPROTEIN HOMOLOG | 7.20E-03 | 1.36 |
| ***IMET 5372*** | PUTATIVESERCA-TYPE CALCIUM ATPASE, PARTIAL | 7.27E-03 | 1.64 |
| ***IMET 9251*** | CONSERVEDUNKNOWN PROTEIN | 7.50E-03 | 1.15 |
| ***IMET 5563*** | NAD-DEPENDENTEPIMERASE DEHYDRATASE | 7.52E-03 | 1.73 |
| ***IMET 3302*** | AQUAPORIN-LIKEPROTEIN | 7.91E-03 | 1.58 |
| ***IMET 4058*** | PREDICTEDPROTEIN | 8.01E-03 | 0.83 |
| ***IMET 1151*** | PREDICTEDPROTEIN | 8.36E-03 | 1.83 |
| ***IMET 3242*** | HYPOTHETICALPROTEIN NGA_0170500 | 8.45E-03 | 1.58 |
| ***IMET 8991*** | CONSERVEDUNKNOWN PROTEIN | 8.60E-03 | 0.42 |
| ***IMET 3235*** | SMALLSUBUNIT RIBOSOMAL PROTEIN S9E | 8.64E-03 | 0.76 |
| ***IMET 7611*** | BETA-ASPARTYL ASPARAGINYL FAMILY | 8.71E-03 | 1.99 |
| ***IMET 9755*** | PHOTOSYSTEMI P700 CHLOROPHYLL A APOPROTEIN A1 | 8.78E-03 | 1.61 |
| ***IMET 4425*** | PEPTIDYL-PROLYLCIS-TRANS ISOMERASE CYP20-1 | 9.04E-03 | 1.69 |
| ***IMET 901*** | PYRUVATEDEHYDROGENASE E2 COMPONENT | 9.25E-03 | 1.95 |
| ***IMET 4381*** | ATP/ADP TRANSLOCATOR | 9.47E-03 | 1.24 |
| ***IMET 2516*** | HYPOTHETICALPROTEIN AURANDRAFT_28296, PARTIAL | 9.57E-03 | 0.47 |
| ***IMET 4944*** | CHLOROPLASTATPASE GAMMA SUBUNIT PRECURSOR | 9.79E-03 | 0.83 |
| ***IMET 438*** | NICOTINAMIDENUCLEOTIDE TRANSHYDROGENASE | 1.00E-02 | 1.16 |
| ***IMET 3478*** | HOMOSERINEDEHYDROGENASE | 1.01E-02 | 1.37 |
| ***IMET 6388*** | CONSERVEDUNKNOWN PROTEIN | 1.02E-02 | 1.59 |
| ***IMET 9697*** | GLYCERALDEHYDE-3-PHOSPHATEDEHYDROGENASE | 1.07E-02 | 0.61 |
| ***IMET 5241*** | CYTOCHROMEB-C1 COMPLEX SUBUNIT RIESKE, PUTATIVE | 1.10E-02 | 2.12 |
| ***IMET*** ***6398*** | HYPOTHETICALPROTEIN THAPSDRAFT_27892 | 1.11E-02 | 2.57 |
| ***IMET 7808*** | PROTEASOMESUBUNIT ALPHA TYPE 7 | 1.13E-02 | 0.86 |
| ***IMET 103*** | ALANYL-TRNASYNTHETASE | 1.16E-02 | 1.08 |
| ***IMET 7304*** | MALATEDEHYDROGENASE | 1.17E-02 | 0.52 |
| ***IMET 5882*** | CONSERVEDUNKNOWN PROTEIN | 1.27E-02 | 1.88 |
| ***IMET 5600*** | PREMRNASPLICINGFACTOR SF2 PUTATIVE | 1.29E-02 | 0.49 |
| ***IMET 4946*** | HYPOTHETICALPROTEIN AURANDRAFT_19845 | 1.32E-02 | 1.57 |
| ***IMET 7897*** | ISOCITRATELYASE | 1.34E-02 | -1.41 |
| ***IMET 4636*** | CONSERVEDUNKNOWN PROTEIN | 1.35E-02 | 0.85 |
| ***IMET 4592*** | NITRICOXIDE DIOXYGENASE | 1.35E-02 | 1.07 |
| ***IMET 5367*** | PREDICTEDPROTEIN | 1.40E-02 | 1.47 |
| ***IMET 4330*** | PEPTIDYL-PROLYLCIS-TRANS ISOMERASE | 1.47E-02 | 0.69 |
| ***IMET 5543*** | SHORT-CHAINDEHYDROGENASE REDUCTASE SDR | 1.49E-02 | 1.19 |
| ***IMET 1767*** | PREDICTEDPROTEIN | 1.49E-02 | -0.95 |
| ***IMET 8745*** | EXPORTIN1 | 1.49E-02 | 2.18 |
| ***IMET 5512*** | HYPOTHETICALPROTEIN PHYSODRAFT_333950 | 1.52E-02 | 0.83 |
| ***IMET 704*** | PREDICTEDPROTEIN | 1.55E-02 | 1.92 |
| ***IMET 7014*** | HYPOTHETICALPROTEIN CHLNCDRAFT_144365 | 1.56E-02 | 1.04 |
| ***IMET 2216*** | HYPOTHETICALPROTEIN NGA_0428900, PARTIAL | 1.56E-02 | 1.41 |
| ***IMET 2396*** | ADENYLATEKINASE, PUTATIVE | 1.60E-02 | 0.79 |
| ***IMET 5917*** | HYPOTHETICALPROTEIN NGA_0713600 | 1.60E-02 | -2.01 |
| ***IMET 7298*** | HYPOTHETICALPROTEIN AURANDRAFT_60075 | 1.62E-02 | 2.32 |
| ***IMET 5167*** | INORGANICPYROPHOSPHATASE | 1.62E-02 | 1.20 |
| ***IMET 2271*** | VCP1 | 1.63E-02 | 0.88 |
| ***IMET 9800*** | 50SRIBOSOMAL PROTEIN L4 | 1.67E-02 | 1.49 |
| ***IMET 9763*** | 50SRIBOSOMAL PROTEIN L12 | 1.70E-02 | 0.82 |
| ***IMET 106*** | SIGNALPEPTIDE PEPTIDASE | 1.71E-02 | 2.10 |
| ***IMET 7054*** | EXPRESSEDUNKNOWN PROTEIN | 1.73E-02 | 1.14 |
| ***IMET 6463*** | HYPOTHETICALPROTEIN AURANDRAFT_22992 | 1.77E-02 | 1.38 |
| ***IMET 9855*** | PHOTOSYSTEMII PROTEIN D1 | 1.81E-02 | 1.59 |
| ***IMET 3202*** | (1,3)-BETA-D-GLUCANBINDING PROTEIN | 1.95E-02 | 1.19 |
| ***IMET 5267*** | HYPOTHETICALPROTEIN AURANDRAFT_37329 | 2.02E-02 | 1.90 |
| ***IMET 9878*** | CLPPROTEASE ATP BINDING SUBUNIT | 2.03E-02 | 1.25 |
| ***IMET 3140*** | HEATSHOCK PROTEIN HSP90 | 2.15E-02 | 0.88 |
| ***IMET 1841*** | SERINEHYDROXYMETHYLTRANSFERASE | 2.19E-02 | 1.03 |
| ***IMET 2203*** | HYPOTHETICALPROTEIN AURANDRAFT_36266 | 2.24E-02 | 1.76 |
| ***IMET 3805*** | HYPOTHETICALPROTEIN RO3G_05085 | 2.28E-02 | 1.25 |
| ***IMET 1829*** | HYPOTHETICALPROTEIN | 2.33E-02 | 1.22 |
| ***IMET 3007*** | PEROXIREDOXIN-LIKEPROTEIN | 2.34E-02 | 1.90 |
| ***IMET 3679*** | ASPARTATE-SEMIALDEHYDEDEHYDROGENASE | 2.41E-02 | 1.44 |
| ***IMET 613*** | GLYCOLATEOXIDASE | 2.42E-02 | 0.66 |
| ***IMET 6185*** | KELCHREPEAT PROTEIN, PARTIAL | 2.43E-02 | 1.67 |
| ***IMET 9491*** | DIHYDROLIPOAMIDEDEHYDROGENASE | 2.49E-02 | 1.09 |
| ***IMET 755*** | RRNAMETHYLTRANSFERASE | 2.53E-02 | 0.68 |
| ***IMET 2789*** | INORGANICPYROPHOSPHATASE | 2.57E-02 | 2.16 |
| ***IMET 5090*** | H+-TRANSPORTINGATPASE | 2.58E-02 | 2.76 |
| ***IMET 4307*** | HYPOTHETICALPROTEIN THAOC_03076 | 2.66E-02 | -0.86 |
| ***IMET 5161*** | CONSERVEDUNKNOWN PROTEIN | 2.67E-02 | 1.61 |
| ***IMET 9849*** | ATPSYNTHASE CF1 EPSILON SUBUNIT | 2.70E-02 | 1.23 |
| ***IMET 9851*** | CYTOCHROMEF | 2.74E-02 | 1.25 |
| ***IMET 2414*** | MITOCHONDRIALP5CDH | 2.84E-02 | 0.70 |
| ***IMET 9900*** | NADHDEHYDROGENASE SUBUNIT 9 | 2.85E-02 | -0.61 |
| ***IMET 5876*** | SIGMA54 MODULATION PROTEIN RIBOSOMAL PROTEIN S30EA | 2.88E-02 | 0.89 |
| ***IMET 178*** | NUCLEOSIDEDIPHOSPHATE KINASE B PUTATIVE | 2.91E-02 | 0.72 |
| ***IMET 1699*** | HYPOTHETICALPROTEIN NGA_0401010 | 2.92E-02 | 0.64 |
| ***IMET 1991*** | HYPOTHETICALPROTEIN NGA_0547200 | 2.94E-02 | 1.65 |
| ***IMET 2832*** | RHD3A,RHD3/SEY1 FAMILY GTPASE INVOLVED IN THE ER-TO-GOLGI TRAFFIC | 3.08E-02 | 1.35 |
| ***IMET 4778*** | FTSZ | 3.13E-02 | 0.82 |
| ***IMET 9405*** | CONSERVEDUNKNOWN PROTEIN | 3.16E-02 | 1.22 |
| ***IMET 8105*** | S-ADENOSYLMETHIONINEMITOCHONDRIAL CARRIER PROTEIN | 3.17E-02 | 1.47 |
| ***IMET 3323*** | H+-TRANSPORTINGATPASE | 3.25E-02 | 1.20 |
| ***IMET 7969*** | PCNA-LIKEPROTEIN | 3.30E-02 | 1.26 |
| ***IMET 102*** | RAB1FAMILY GTPASE | 3.45E-02 | 1.08 |
| ***IMET 9205*** | PYRUVATEKINASE, PUTATIVE | 3.51E-02 | -1.47 |
| ***IMET 5283*** | ALPHATUBULIN | 3.66E-02 | 1.80 |
| ***IMET 865*** | LIGHTHARVESTING COMPLEX PROTEIN | 3.68E-02 | 1.00 |
| ***IMET 1570*** | HYPOTHETICALPROTEIN | 3.74E-02 | 2.28 |
| ***IMET 977*** | RIBONUCLEASEP | 3.82E-02 | 1.22 |
| ***IMET 8365*** | ACETYL-CO-ACARBOXYLASE | 3.83E-02 | -0.37 |
| ***IMET 342*** | ETHYLMALONICENCEPHALOPATHY 1 | 3.83E-02 | 1.66 |
| ***IMET 4248*** | CONSERVEDUNKNOWN PROTEIN | 3.95E-02 | 1.10 |
| ***IMET 4196*** | RABGDP DISSOCIATION INHIBITOR ALPHA PUTATIVE | 3.95E-02 | 1.48 |
| ***IMET 9812*** | PHOTOSYSTEMI SUBUNIT III | 4.04E-02 | 1.69 |
| ***IMET 8393*** | PAPFIBRILLIN FAMILY PROTEIN | 4.21E-02 | -0.93 |
| ***IMET 3033*** | HEATSHOCK PROTEIN 70 | 4.27E-02 | -0.60 |
| ***IMET 9695*** | RECNAME:FULL=CALMODULIN; SHORT=CAM &GT;GI | 4.36E-02 | 1.45 |
| ***IMET 228*** | LIGHT-HARVESTINGPROTEIN | 4.36E-02 | 0.99 |
| ***IMET 6281*** | IMPORTINBETA SUBUNIT | 4.39E-02 | 1.11 |
| ***IMET 4088*** | CYCLOPHILIN-LIKEPROTEIN | 4.48E-02 | 2.91 |
| ***IMET 1469*** | HYPOTHETICALPROTEIN AURANDRAFT_28033 | 4.51E-02 | 1.32 |
| ***IMET 6737*** | HYPOTHETICALPROTEIN SORBIDRAFT_04G033900 | 4.62E-02 | 2.48 |
| ***IMET 7454*** | ATP-DEPENDENTCLP PROTEASE PROTEOLYTIC SUBUNIT | 4.72E-02 | 0.97 |
| ***IMET 3060*** | TRANSITIONALENDOPLASMIC RETICULUM ATPASE | 4.73E-02 | 1.73 |
| ***IMET 5506*** | LONG-CHAINACYL-COA LIGASE | 4.76E-02 | 0.87 |
| ***IMET 9877*** | 50SRIBOSOMAL PROTEIN L19 | 4.95E-02 | 0.41 |
| ***IMET 1900*** | HYPOTHETICALPROTEIN AURANDRAFT_37947 | 4.98E-02 | -0.96 |

# Reference

1. Chen W, Zhang C, Song L, Sommerfeld M, Hu Q: **A high throughput Nile red method for quantitative measurement of neutral lipids in microalgae**. *Journal of Microbiological Methods* 2015, **77**(1):41-47.

2. Tran NAT, Padula MP, Evenhuis CR, Commault AS, Ralph PJ, Tamburic B: **Proteomic and biophysical analyses reveal a metabolic shift in nitrogen deprived Nannochloropsis oculata**. *Algal Research* 2016, **19**:1-11.
